# Supplementary material for: Performance of 11 Host Biomarkers Alone or in Combination in the Diagnosis of Late-Onset Sepsis in Hospitalized Neonates: The Prospective EMERAUDE Study
Source: Biomedicines. 2023 Jun 13;11(6):1703. doi: 10.3390/biomedicines11061703 (PMC10295850; doi:10.3390/biomedicines11061703)
Supplement: Supplementary file 1 [file biomedicines-11-01703-s001.zip › Supplementary Material.pdf]

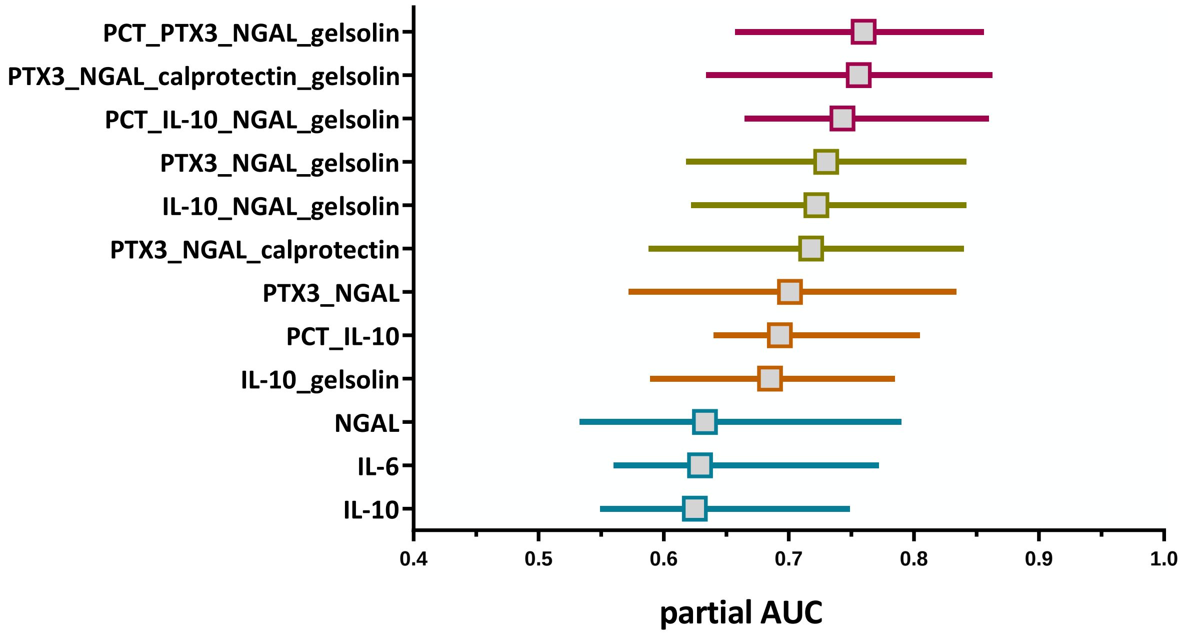

Figure S1. Performance of best performing models for biomarker alone or in combination for infection diagnosis. The forest plots depict partial AUC (plain circles) and respective 95% CI (horizontal whiskers). The 3 best partial AUC are represented for models with a combination of 1 to 4 biomarkers. The squares represent partial AUC and bars indicate the 95% confidence interval.

Table S1. Biomarker concentration according to infection status.

	All patients (N=230)	Infected (N=51)	Not Infected (N=153)	Unclassified (N=26)	*P* value[a] (Infected vs Not Infected)	CutOff[b]
**IL-6** (pg/mL) N		51	153	26		4.74 pg/mL
**IL-6** (pg/mL) median (range)	9.2 (0.3 - 63292.0)	105 (1.61 - 63292)	5.52 (0.3 - 963)	37.3 (2.34 - 4662)	<.001	
**PCT** (pg/mL) N		51	153	26		510 pg/mL
**PCT** (pg/mL) median (range)	1524.0 (137.0 - 24472.0)	2392 (226 - 24472)	1237 (137 - 8396)	1867.5 (479 - 18962)	<.001	
**IP-10** (pg/mL) N		51	153	26		77.7 pg/mL
**IP-10** (pg/mL) median (range)	114.5 (17.7 - 5876.0)	218 (29.2 - 5876)	103 (17.7 - 1299)	180 (39.6 - 2542)	<.001	
**IL-10** (pg/mL) N		51	153	26		7.27 pg/mL
**IL-10** (pg/mL) median (range)	8.4 (2.5 - 3861.0)	53.2 (4.4 - 3861)	7.13 (2.48 - 158)	13.5 (4.55 - 1737)	<.001	
**PTX3** (pg/mL) N		49	153	26		1266 pg/mL
**PTX3** (pg/mL) (n= 228) median (range)	2416.0 (427.0 - 150366)	3951 (965 - 150366)	1856 (427 - 14196)	4253 (1429 - 25892)	<.001	
**CD14** (ng/mL) N		49	153	26		712.2 ng/mL
**CD14** (ng/mL) (n= 228) median (range)	887.0 (372.5 - 3617.3)	1163.2 (491.2 - 3617.3)	800.3 (372.5 - 2222.2)	1093.95 (504.3 - 2185)	<.001	
**LBP** (ng/mL) N		49	153	26		6172 ng/mL
**LBP** (ng/mL) (n= 228) median (range)	9369.0 (2142.0 - 70715.0)	18783 (2866 - 70715)	7252 (2142 - 64445)	19608.5 (4079 - 51649)	<.001	
**NGAL** (ng/mL) N		49	153	26		95.9 ng/mL
**NGAL** (ng/mL) (n= 228) median (range)	101.7 (30.1 - 1342.6)	205.5 (44.8 - 1342.6)	79.7 (30.1 - 451.7)	159.45 (34.7 - 1119.9)	<.001	
**calprotectin** (ng/mL) N		51	150	26		748.1 ng/mL
**calprotectin** (ng/mL) (n= 227) median (range)	1587.5 (150.5 - 29815.0)	2702.4 (477.6 - 22845)	1302.2 (150.5 - 29815)	2095.2 (241.2 - 24565)	<.001	
**gelsolin** (µg/mL) N		49	150	26		133.3 µg/mL
**gelsolin** (µg/mL) (n= 225) median (range)	207.3 (69.9 - 1595.9)	209.3 (69.9 - 1106.3)	203.45 (90.5 - 1595.9)	236.65 (82.6 - 1278.2)	NS	
**IL-27** (pg/mL) N		27	95	14		
**IL-27** (pg/mL) (n= 136) median (range)	312.2 (0.0 - 101063)	485.1 (107.2 - 20996.1)	266.4 (0 - 11228.6)	254.65 (0 - 101063)	NA	NA

[a]*P* value was calculated using Kruskal-Wallis test. NS non-significant. NA not available.

[b]The Cut-off with the highest specificity and a sensitivity of ≥0.898 was estimated for each biomarker. NA not available.

Table S2. Performances of biomarker combinations using logistic regression for infection diagnosis.

Model	N	AUC [95% CI]	AUC*	Partial AUC	Partial AUC*	Sensitivity	Specificity	NPV	PPV	LR+	LR-
NGAL	202	0.829 [0.760-0.898]	0.828	0.633 [0.533-0.790]	0.579	0.898	0.614 [0.532-0.692]	0.949 [0.886-0.983]	0.427 [0.330-0.528]	2.329 [1.867-2.905]	0.166 [0.072-0.385]
IL-6	204	0.864 [0.798-0.929]	0.862	0.629 [0.560-0.772]	0.566	0.902	0.438 [0.358-0.520]	0.931 [0.845-0.977]	0.348 [0.268-0.436]	1.605 [1.358-1.896]	0.224 [0.096-0.525]
IL-10	204	0.845 [0.777-0.914]	0.843	0.625 [0.549-0.749]	0.588	0.902	0.536 [0.454-0.617]	0.943 [0.871-0.981]	0.393 [0.304-0.488]	1.944 [1.603-2.357]	0.183 [0.079-0.426]
PTX3	202	0.732 [0.652-0.811]	0.726	0.588 [0.555-0.670]	0.547	0.898	0.294 [0.223-0.373]	0.900 [0.782-0.967]	0.289 [0.219-0.368]	1.272 [1.107-1.462]	0.347 [0.146-0.825]
calprotectin	201	0.690 [0.609-0.772]	0.689	0.583 [0.538-0.662]	0.557	0.902	0.287 [0.216-0.366]	0.896 [0.773-0.965]	0.301 [0.229-0.380]	1.264 [1.104-1.449]	0.342 [0.143-0.816]
LBP	202	0.786 [0.707-0.865]	0.787	0.576 [0.519-0.695]	0.497	0.898	0.346 [0.271-0.427]	0.914 [0.810-0.971]	0.306 [0.232-0.388]	1.374 [1.184-1.595]	0.295 [0.125-0.695]
IP-10	204	0.732 [0.644-0.821]	0.736	0.521 [0.488-0.630]	0.433	0.902	0.268 [0.200-0.345]	0.891 [0.764-0.964]	0.291 [0.222-0.369]	1.232 [1.080-1.406]	0.366 [0.153-0.876]
CD14	202	0.734 [0.647-0.821]	0.737	0.506 [0.486-0.658]	0.423	0.898	0.340 [0.265-0.421]	0.912 [0.807-0.971]	0.303 [0.230-0.385]	1.360 [1.173-1.577]	0.300 [0.127-0.709]
PCT	204	0.666 [0.568-0.763]	0.668	0.502 [0.481-0.548]	0.491	0.902	0.137 [0.087-0.202]	0.808 [0.606-0.934]	0.258 [0.196-0.329]	1.045 [0.936-1.167]	0.714 [0.284-1.797]
gelsolin	199	0.537 [0.440-0.634]	0.525		.	0.898	0.073 [0.037-0.127]	0.688 [0.413-0.890]	0.240 [0.180-0.309]	0.969 [0.873-1.076]	1.391 [0.508-3.808]
PTX3 NGAL	202	0.845 [0.783-0.906]	0.832	0.701 [0.572-0.834]	0.686	0.898	0.654 [0.573-0.729]	0.952 [0.892-0.984]	0.454 [0.352-0.558]	2.592 [2.045-3.286]	0.156 [0.067-0.361]
PCT IL-10	204	0.871 [0.814-0.927]	0.865	0.693 [0.640-0.805]	0.667	0.902	0.660 [0.579-0.735]	0.953 [0.893-0.985]	0.469 [0.368-0.573]	2.654 [2.090-3.369]	0.149 [0.064-0.344]
IL-10 gelsolin	199	0.862 [0.799-0.925]	0.858	0.685 [0.589-0.785]	0.662	0.898	0.547 [0.463-0.628]	0.943 [0.871-0.981]	0.393 [0.302-0.490]	1.981 [1.623-2.418]	0.187 [0.080-0.434]
IL-10 PTX3	202	0.864 [0.801-0.928]	0.862	0.682 [0.602-0.764]	0.665	0.898	0.510 [0.428-0.591]	0.940 [0.865-0.980]	0.370 [0.283-0.463]	1.832 [1.519-2.209]	0.200 [0.086-0.466]
IL-10 NGAL	202	0.883 [0.824-0.943]	0.875	0.682 [0.565-0.829]	0.642	0.898	0.654 [0.573-0.729]	0.952 [0.892-0.984]	0.454 [0.352-0.558]	2.592 [2.045-3.286]	0.156 [0.067-0.361]
IL-6 PCT	204	0.879 [0.820-0.938]	0.875	0.674 [0.601-0.795]	0.635	0.902	0.562 [0.480-0.642]	0.945 [0.876-0.982]	0.407 [0.316-0.504]	2.060 [1.685-2.518]	0.174 [0.075-0.406]
IL-6 PTX3	202	0.870 [0.808-0.932]	0.865	0.670 [0.602-0.774]	0.634	0.898	0.562 [0.480-0.642]	0.945 [0.876-0.982]	0.396 [0.305-0.494]	2.051 [1.674-2.512]	0.182 [0.078-0.421]
NGAL gelsolin	199	0.845 [0.782-0.909]	0.844	0.669 [0.572-0.807]	0.637	0.898	0.627 [0.544-0.704]	0.949 [0.886-0.983]	0.440 [0.341-0.543]	2.405 [1.915-3.021]	0.163 [0.070-0.377]
IL-10 LBP	202	0.873 [0.811-0.935]	0.867	0.667 [0.608-0.779]	0.646	0.898	0.549 [0.467-0.629]	0.944 [0.874-0.982]	0.389 [0.299-0.486]	1.991 [1.632-2.429]	0.186 [0.080-0.432]
IL-6 NGAL	202	0.875 [0.813-0.937]	0.869	0.664 [0.573-0.804]	0.634	0.898	0.614 [0.532-0.692]	0.949 [0.886-0.983]	0.427 [0.330-0.528]	2.329 [1.867-2.905]	0.166 [0.072-0.385]
LBP NGAL	202	0.839 [0.772-0.906]	0.838	0.649 [0.558-0.792]	0.626	0.898	0.627 [0.546-0.704]	0.950 [0.888-0.984]	0.436 [0.337-0.538]	2.410 [1.922-3.022]	0.163 [0.070-0.377]
IL-10 CD14	202	0.864 [0.798-0.929]	0.860	0.639 [0.562-0.788]	0.598	0.898	0.595 [0.513-0.673]	0.948 [0.883-0.983]	0.415 [0.320-0.515]	2.216 [1.789-2.744]	0.172 [0.074-0.398]
IL-6 calprotectin	201	0.860 [0.795-0.925]	0.855	0.638 [0.568-0.761]	0.594	0.902	0.473 [0.391-0.556]	0.934 [0.853-0.978]	0.368 [0.284-0.459]	1.713 [1.435-2.043]	0.207 [0.089-0.484]
PCT NGAL	202	0.830 [0.762-0.899]	0.819	0.637 [0.535-0.795]	0.592	0.898	0.621 [0.539-0.698]	0.950 [0.887-0.984]	0.431 [0.334-0.533]	2.369 [1.894-2.963]	0.164 [0.071-0.381]
IL-6 IL-10	204	0.879 [0.817-0.940]	0.872	0.637 [0.564-0.805]	0.586	0.902	0.582 [0.499-0.661]	0.947 [0.880-0.983]	0.418 [0.325-0.516]	2.156 [1.752-2.654]	0.169 [0.073-0.392]
PTX3 calprotectin	199	0.761 [0.688-0.834]	0.752	0.635 [0.559-0.728]	0.599	0.898	0.460 [0.378-0.543]	0.932 [0.849-0.978]	0.352 [0.269-0.442]	1.663 [1.396-1.981]	0.222 [0.095-0.518]
IL-6 CD14	202	0.858 [0.791-0.926]	0.854	0.632 [0.558-0.752]	0.579	0.898	0.425 [0.345-0.507]	0.929 [0.841-0.976]	0.333 [0.254-0.421]	1.561 [1.323-1.843]	0.240 [0.103-0.562]
PTX3 LBP	202	0.812 [0.742-0.883]	0.807	0.630 [0.554-0.749]	0.587	0.898	0.510 [0.428-0.591]	0.940 [0.865-0.980]	0.370 [0.283-0.463]	1.832 [1.519-2.209]	0.200 [0.086-0.466]
NGAL calprotectin	199	0.827 [0.757-0.897]	0.823	0.627 [0.523-0.792]	0.576	0.898	0.620 [0.537-0.698]	0.949 [0.885-0.983]	0.436 [0.337-0.538]	2.363 [1.887-2.960]	0.165 [0.071-0.381]
PCT PTX3	202	0.751 [0.676-0.827]	0.739	0.627 [0.558-0.727]	0.603	0.898	0.484 [0.402-0.566]	0.937 [0.858-0.979]	0.358 [0.273-0.449]	1.739 [1.452-2.082]	0.211 [0.090-0.492]
IL-6 gelsolin	199	0.861 [0.795-0.928]	0.860	0.626 [0.565-0.768]	0.571	0.898	0.487 [0.404-0.570]	0.936 [0.857-0.979]	0.364 [0.278-0.456]	1.749 [1.458-2.099]	0.210 [0.090-0.489]

IP-10 IL-10	204	0.846 [0.777-0.914]	0.839	0.626 [0.548-0.748]	0.599	0.902	0.536 [0.454-0.617]	0.943 [0.871-0.981]	0.393 [0.304-0.488]	1.944 [1.603-2.357]	0.183 [0.079-0.426]
IL-6 LBP	202	0.861 [0.792-0.929]	0.856	0.624 [0.548-0.755]	0.542	0.898	0.431 [0.352-0.514]	0.930 [0.843-0.977]	0.336 [0.256-0.424]	1.579 [1.336-1.867]	0.237 [0.101-0.554]
IL-10 calprotectin	201	0.845 [0.775-0.915]	0.841	0.618 [0.534-0.736]	0.563	0.902	0.480 [0.398-0.563]	0.935 [0.855-0.979]	0.371 [0.286-0.462]	1.735 [1.451-2.073]	0.204 [0.087-0.477]
PCT calprotectin	201	0.733 [0.657-0.808]	0.719	0.617 [0.565-0.712]	0.59	0.902	0.360 [0.283-0.442]	0.915 [0.813-0.972]	0.324 [0.248-0.408]	1.409 [1.213-1.638]	0.272 [0.115-0.643]
IP-10 NGAL	202	0.846 [0.778-0.915]	0.842	0.616 [0.512-0.797]	0.533	0.898	0.647 [0.566-0.723]	0.952 [0.891-0.984]	0.449 [0.348-0.553]	2.544 [2.013-3.216]	0.158 [0.068-0.365]
PTX3 CD14	202	0.785 [0.710-0.859]	0.781	0.616 [0.546-0.720]	0.571	0.898	0.444 [0.364-0.527]	0.932 [0.847-0.977]	0.341 [0.260-0.430]	1.616 [1.363-1.916]	0.230 [0.098-0.537]
IL-6 IP-10	204	0.862 [0.796-0.929]	0.857	0.612 [0.551-0.773]	0.552	0.902	0.412 [0.333-0.494]	0.926 [0.837-0.976]	0.338 [0.259-0.424]	1.533 [1.306-1.800]	0.238 [0.101-0.559]
CD14 NGAL	202	0.831 [0.761-0.901]	0.823	0.610 [0.521-0.784]	0.555	0.898	0.627 [0.546-0.704]	0.950 [0.888-0.984]	0.436 [0.337-0.538]	2.410 [1.922-3.022]	0.163 [0.070-0.377]
IP-10 calprotectin	201	0.767 [0.691-0.843]	0.759	0.607 [0.527-0.720]	0.563	0.902	0.413 [0.334-0.497]	0.925 [0.834-0.975]	0.343 [0.263-0.430]	1.537 [1.308-1.808]	0.237 [0.101-0.557]
LBP calprotectin	199	0.800 [0.726-0.875]	0.800	0.605 [0.534-0.731]	0.522	0.898	0.460 [0.378-0.543]	0.932 [0.849-0.978]	0.352 [0.269-0.442]	1.663 [1.396-1.981]	0.222 [0.095-0.518]
IP-10 PTX3	202	0.787 [0.710-0.864]	0.780	0.604 [0.537-0.699]	0.569	0.898	0.405 [0.327-0.487]	0.925 [0.834-0.975]	0.326 [0.248-0.412]	1.510 [1.285-1.774]	0.252 [0.107-0.591]
PTX3 gelsolin	199	0.732 [0.652-0.812]	0.719	0.596 [0.562-0.669]	0.559	0.898	0.287 [0.216-0.366]	0.896 [0.773-0.965]	0.291 [0.220-0.371]	1.259 [1.096-1.446]	0.356 [0.149-0.848]
calprotectin gelsolin	199	0.703 [0.622-0.784]	0.690	0.595 [0.553-0.676]	0.577	0.898	0.327 [0.252-0.408]	0.907 [0.797-0.969]	0.303 [0.230-0.385]	1.334 [1.152-1.543]	0.312 [0.132-0.740]
PCT LBP	202	0.798 [0.721-0.875]	0.789	0.587 [0.494-0.720]	0.488	0.898	0.392 [0.314-0.474]	0.923 [0.830-0.975]	0.321 [0.244-0.406]	1.477 [1.261-1.731]	0.260 [0.111-0.611]
PCT IP-10	204	0.782 [0.705-0.858]	0.774	0.586 [0.497-0.708]	0.511	0.902	0.405 [0.327-0.487]	0.925 [0.834-0.975]	0.336 [0.257-0.421]	1.516 [1.294-1.778]	0.242 [0.103-0.568]
CD14 LBP	202	0.792 [0.714-0.870]	0.784	0.575 [0.513-0.709]	0.499	0.898	0.464 [0.383-0.546]	0.934 [0.853-0.978]	0.349 [0.266-0.439]	1.675 [1.406-1.996]	0.220 [0.094-0.513]
LBP gelsolin	199	0.784 [0.704-0.864]	0.778	0.574 [0.520-0.694]	0.5	0.898	0.333 [0.259-0.415]	0.909 [0.800-0.970]	0.306 [0.232-0.388]	1.347 [1.162-1.561]	0.306 [0.129-0.724]
IP-10 CD14	202	0.788 [0.705-0.870]	0.785	0.563 [0.484-0.685]	0.47	0.898	0.386 [0.308-0.468]	0.922 [0.827-0.974]	0.319 [0.242-0.404]	1.462 [1.249-1.710]	0.265 [0.113-0.622]
CD14 calprotectin	199	0.759 [0.678-0.840]	0.750	0.562 [0.530-0.687]	0.515	0.898	0.267 [0.198-0.345]	0.889 [0.759-0.963]	0.286 [0.216-0.364]	1.224 [1.070-1.401]	0.383 [0.160-0.915]
IP-10 LBP	202	0.814 [0.734-0.893]	0.809	0.560 [0.498-0.696]	0.456	0.898	0.392 [0.314-0.474]	0.923 [0.830-0.975]	0.321 [0.244-0.406]	1.477 [1.261-1.731]	0.260 [0.111-0.611]
PCT CD14	202	0.756 [0.670-0.842]	0.744	0.527 [0.488-0.663]	0.443	0.898	0.176 [0.120-0.246]	0.844 [0.672-0.947]	0.259 [0.195-0.331]	1.090 [0.968-1.229]	0.578 [0.235-1.420]
IP-10 gelsolin	199	0.728 [0.636-0.819]	0.721	0.514 [0.486-0.622]	0.464	0.898	0.280 [0.210-0.359]	0.894 [0.769-0.965]	0.289 [0.219-0.368]	1.247 [1.087-1.431]	0.364 [0.153-0.869]
CD14 gelsolin	199	0.737 [0.650-0.824]	0.725	0.509 [0.488-0.660]	0.417	0.898	0.367 [0.290-0.449]	0.917 [0.816-0.972]	0.317 [0.240-0.401]	1.418 [1.215-1.654]	0.278 [0.118-0.656]
PCT gelsolin	199	0.658 [0.558-0.758]	0.649		.	0.898	0.127 [0.078-0.191]	0.792 [0.578-0.929]	0.251 [0.189-0.322]	1.028 [0.919-1.150]	0.806 [0.318-2.043]
PTX3 NGAL gelsolin	199	0.856 [0.800-0.913]	0.840	0.730 [0.618-0.842]	0.726	0.898	0.640 [0.558-0.717]	0.950 [0.888-0.984]	0.449 [0.348-0.553]	2.494 [1.975-3.150]	0.159 [0.069-0.369]
IL-10 NGAL gelsolin	199	0.894 [0.841-0.947]	0.883	0.722 [0.622-0.842]	0.701	0.898	0.707 [0.627-0.778]	0.955 [0.898-0.985]	0.500 [0.391-0.609]	3.061 [2.347-3.993]	0.144 [0.063-0.333]
PTX3 NGAL calprotectin	199	0.849 [0.790-0.909]	0.834	0.718 [0.588-0.840]	0.708	0.898	0.673 [0.592-0.748]	0.953 [0.893-0.985]	0.473 [0.369-0.579]	2.749 [2.144-3.524]	0.152 [0.066-0.350]
PCT PTX3 NGAL	202	0.846 [0.786-0.907]	0.824	0.708 [0.582-0.833]	0.695	0.898	0.647 [0.566-0.723]	0.952 [0.891-0.984]	0.449 [0.348-0.553]	2.544 [2.013-3.216]	0.158 [0.068-0.365]
IL-10 PTX3 NGAL	202	0.885 [0.829-0.942]	0.877	0.704 [0.604-0.825]	0.686	0.898	0.647 [0.566-0.723]	0.952 [0.891-0.984]	0.449 [0.348-0.553]	2.544 [2.013-3.216]	0.158 [0.068-0.365]
PCT IL-10 PTX3	202	0.879 [0.822-0.936]	0.874	0.701 [0.638-0.796]	0.682	0.898	0.608 [0.526-0.686]	0.949 [0.885-0.983]	0.423 [0.327-0.524]	2.290 [1.840-2.850]	0.168 [0.072-0.389]
PCT IL-10 LBP	202	0.883 [0.826-0.940]	0.868	0.701 [0.643-0.800]	0.691	0.898	0.556 [0.473-0.636]	0.944 [0.875-0.982]	0.393 [0.302-0.490]	2.020 [1.653-2.470]	0.184 [0.079-0.427]
IL-10 PTX3 gelsolin	199	0.870 [0.808-0.931]	0.858	0.701 [0.603-0.787]	0.682	0.898	0.533 [0.450-0.615]	0.941 [0.868-0.981]	0.386 [0.296-0.482]	1.924 [1.583-2.339]	0.191 [0.082-0.445]
PCT NGAL gelsolin	199	0.850 [0.790-0.909]	0.838	0.700 [0.600-0.822]	0.687	0.898	0.640 [0.558-0.717]	0.950 [0.888-0.984]	0.449 [0.348-0.553]	2.494 [1.975-3.150]	0.159 [0.069-0.369]
PCT IL-10 NGAL	202	0.888 [0.831-0.945]	0.878	0.696 [0.577-0.839]	0.668	0.898	0.673 [0.593-0.747]	0.954 [0.895-0.985]	0.468 [0.364-0.574]	2.748 [2.148-3.515]	0.152 [0.066-0.350]

IL-6 NGAL gelsolin	199	0.881 [0.824-0.939]	0.871	0.694 [0.621-0.803]	0.677	0.898	0.580 [0.497-0.660]	0.946 [0.878-0.982]	0.411 [0.317-0.510]	2.138 [1.732-2.639]	0.176 [0.076-0.408]
IL-6 PCT PTX3	202	0.882 [0.824-0.939]	0.876	0.694 [0.628-0.795]	0.667	0.898	0.608 [0.526-0.686]	0.949 [0.885-0.983]	0.423 [0.327-0.524]	2.290 [1.840-2.850]	0.168 [0.072-0.389]
PCT IP-10 IL-10	204	0.871 [0.815-0.928]	0.857	0.692 [0.637-0.806]	0.671	0.902	0.660 [0.579-0.735]	0.953 [0.893-0.985]	0.469 [0.368-0.573]	2.654 [2.090-3.369]	0.149 [0.064-0.344]
PTX3 LBP NGAL	202	0.850 [0.789-0.911]	0.834	0.690 [0.587-0.822]	0.684	0.898	0.641 [0.559-0.716]	0.951 [0.890-0.984]	0.444 [0.345-0.548]	2.498 [1.982-3.149]	0.159 [0.069-0.369]
IL-10 CD14 NGAL	202	0.882 [0.823-0.942]	0.872	0.689 [0.566-0.828]	0.659	0.898	0.634 [0.552-0.710]	0.951 [0.889-0.984]	0.440 [0.341-0.543]	2.453 [1.951-3.084]	0.161 [0.070-0.373]
IL-10 LBP gelsolin	199	0.877 [0.818-0.936]	0.865	0.688 [0.625-0.785]	0.667	0.898	0.580 [0.497-0.660]	0.946 [0.878-0.982]	0.411 [0.317-0.510]	2.138 [1.732-2.639]	0.176 [0.076-0.408]
IL-6 PCT gelsolin	199	0.878 [0.819-0.936]	0.873	0.687 [0.645-0.785]	0.661	0.898	0.547 [0.463-0.628]	0.943 [0.871-0.981]	0.393 [0.302-0.490]	1.981 [1.623-2.418]	0.187 [0.080-0.434]
PCT IL-10 gelsolin	199	0.883 [0.827-0.940]	0.870	0.687 [0.624-0.825]	0.66	0.898	0.707 [0.627-0.778]	0.955 [0.898-0.985]	0.500 [0.391-0.609]	3.061 [2.347-3.993]	0.144 [0.063-0.333]
IL-10 PTX3 LBP	202	0.878 [0.819-0.938]	0.871	0.687 [0.627-0.783]	0.678	0.898	0.562 [0.480-0.642]	0.945 [0.876-0.982]	0.396 [0.305-0.494]	2.051 [1.674-2.512]	0.182 [0.078-0.421]
LBP NGAL gelsolin	199	0.850 [0.788-0.912]	0.839	0.685 [0.601-0.805]	0.673	0.898	0.640 [0.558-0.717]	0.950 [0.888-0.984]	0.449 [0.348-0.553]	2.494 [1.975-3.150]	0.159 [0.069-0.369]
IP-10 IL-10 PTX3	202	0.864 [0.800-0.928]	0.858	0.685 [0.605-0.762]	0.671	0.898	0.503 [0.421-0.585]	0.939 [0.863-0.980]	0.367 [0.281-0.459]	1.808 [1.502-2.176]	0.203 [0.087-0.472]
IP-10 IL-10 gelsolin	199	0.863 [0.800-0.926]	0.854	0.685 [0.588-0.790]	0.666	0.898	0.567 [0.483-0.647]	0.944 [0.875-0.982]	0.404 [0.311-0.502]	2.072 [1.687-2.546]	0.180 [0.078-0.418]
IL-6 PCT IL-10	204	0.896 [0.841-0.951]	0.887	0.685 [0.611-0.826]	0.647	0.902	0.569 [0.486-0.648]	0.946 [0.878-0.982]	0.411 [0.319-0.508]	2.091 [1.706-2.562]	0.172 [0.074-0.401]
PTX3 CD14 NGAL	202	0.845 [0.783-0.908]	0.830	0.683 [0.563-0.821]	0.664	0.898	0.634 [0.552-0.710]	0.951 [0.889-0.984]	0.440 [0.341-0.543]	2.453 [1.951-3.084]	0.161 [0.070-0.373]
IL-10 PTX3 CD14	202	0.866 [0.804-0.929]	0.854	0.681 [0.601-0.771]	0.669	0.898	0.536 [0.454-0.617]	0.943 [0.871-0.981]	0.383 [0.294-0.478]	1.935 [1.593-2.351]	0.190 [0.082-0.443]
IP-10 IL-10 NGAL	202	0.882 [0.823-0.942]	0.880	0.680 [0.568-0.824]	0.649	0.898	0.654 [0.573-0.729]	0.952 [0.892-0.984]	0.454 [0.352-0.558]	2.592 [2.045-3.286]	0.156 [0.067-0.361]
IP-10 PTX3 NGAL	202	0.856 [0.793-0.918]	0.842	0.679 [0.537-0.838]	0.62	0.898	0.693 [0.613-0.765]	0.955 [0.898-0.985]	0.484 [0.377-0.591]	2.923 [2.263-3.776]	0.147 [0.064-0.340]
IL-6 PTX3 NGAL	202	0.880 [0.821-0.939]	0.869	0.679 [0.604-0.816]	0.661	0.898	0.634 [0.552-0.710]	0.951 [0.889-0.984]	0.440 [0.341-0.543]	2.453 [1.951-3.084]	0.161 [0.070-0.373]
IL-10 CD14 gelsolin	199	0.871 [0.810-0.932]	0.859	0.679 [0.578-0.814]	0.663	0.898	0.667 [0.585-0.741]	0.952 [0.892-0.984]	0.468 [0.364-0.574]	2.694 [2.108-3.442]	0.153 [0.066-0.354]
IL-6 PCT calprotectin	201	0.876 [0.817-0.935]	0.868	0.677 [0.597-0.790]	0.658	0.902	0.553 [0.470-0.634]	0.943 [0.872-0.981]	0.407 [0.316-0.504]	2.019 [1.654-2.466]	0.177 [0.076-0.412]
IL-10 NGAL calprotectin	199	0.882 [0.822-0.942]	0.870	0.677 [0.562-0.818]	0.643	0.898	0.627 [0.544-0.704]	0.949 [0.886-0.983]	0.440 [0.341-0.543]	2.405 [1.915-3.021]	0.163 [0.070-0.377]
IL-6 PCT NGAL	202	0.882 [0.823-0.942]	0.873	0.676 [0.587-0.814]	0.65	0.898	0.641 [0.559-0.716]	0.951 [0.890-0.984]	0.444 [0.345-0.548]	2.498 [1.982-3.149]	0.159 [0.069-0.369]
IL-10 calprotectin gelsolin	199	0.874 [0.814-0.935]	0.864	0.675 [0.600-0.801]	0.652	0.898	0.553 [0.470-0.634]	0.943 [0.872-0.981]	0.396 [0.305-0.494]	2.010 [1.643-2.459]	0.184 [0.079-0.428]
IL-10 LBP NGAL	202	0.884 [0.824-0.944]	0.874	0.674 [0.577-0.812]	0.661	0.898	0.601 [0.519-0.679]	0.948 [0.884-0.983]	0.419 [0.323-0.519]	2.252 [1.814-2.796]	0.170 [0.073-0.393]
IL-6 PTX3 calprotectin	199	0.866 [0.804-0.929]	0.856	0.673 [0.602-0.771]	0.654	0.898	0.493 [0.411-0.576]	0.937 [0.858-0.979]	0.367 [0.281-0.459]	1.772 [1.474-2.130]	0.207 [0.089-0.482]
IL-10 PTX3 calprotectin	199	0.864 [0.800-0.927]	0.855	0.673 [0.625-0.758]	0.656	0.898	0.467 [0.385-0.550]	0.933 [0.851-0.978]	0.355 [0.271-0.446]	1.684 [1.411-2.010]	0.219 [0.094-0.511]
PCT IL-10 calprotectin	201	0.868 [0.808-0.928]	0.859	0.671 [0.620-0.784]	0.651	0.902	0.533 [0.450-0.615]	0.941 [0.868-0.981]	0.397 [0.307-0.492]	1.933 [1.593-2.345]	0.184 [0.079-0.428]
IL-6 PTX3 gelsolin	199	0.871 [0.810-0.932]	0.862	0.671 [0.609-0.780]	0.648	0.898	0.593 [0.510-0.673]	0.947 [0.880-0.983]	0.419 [0.323-0.519]	2.208 [1.781-2.738]	0.172 [0.074-0.399]
IL-6 IL-10 PTX3	202	0.883 [0.823-0.942]	0.873	0.670 [0.596-0.801]	0.647	0.898	0.588 [0.506-0.667]	0.947 [0.881-0.983]	0.411 [0.317-0.510]	2.181 [1.765-2.695]	0.173 [0.075-0.402]
PCT IL-10 CD14	202	0.879 [0.820-0.938]	0.871	0.670 [0.614-0.811]	0.638	0.898	0.588 [0.506-0.667]	0.947 [0.881-0.983]	0.411 [0.317-0.510]	2.181 [1.765-2.695]	0.173 [0.075-0.402]
IP-10 IL-10 LBP	202	0.874 [0.812-0.935]	0.869	0.670 [0.608-0.780]	0.659	0.898	0.542 [0.460-0.623]	0.943 [0.872-0.981]	0.386 [0.296-0.482]	1.963 [1.612-2.389]	0.188 [0.081-0.437]
IL-6 IL-10 gelsolin	199	0.885 [0.824-0.945]	0.875	0.668 [0.578-0.820]	0.623	0.898	0.520 [0.437-0.602]	0.940 [0.865-0.980]	0.379 [0.291-0.474]	1.871 [1.545-2.265]	0.196 [0.084-0.457]
IL-6 CD14 NGAL	202	0.875 [0.814-0.937]	0.868	0.667 [0.575-0.803]	0.644	0.898	0.608 [0.526-0.686]	0.949 [0.885-0.983]	0.423 [0.327-0.524]	2.290 [1.840-2.850]	0.168 [0.072-0.389]
NGAL calprotectin gelsolin	199	0.844 [0.781-0.908]	0.839	0.667 [0.567-0.810]	0.638	0.898	0.647 [0.565-0.723]	0.951 [0.889-0.984]	0.454 [0.352-0.558]	2.541 [2.007-3.218]	0.158 [0.068-0.365]

IL-10 LBP calprotectin	199	0.870 [0.806-0.933]	0.860	0.666 [0.619-0.760]	0.639	0.898	0.460 [0.378-0.543]	0.932 [0.849-0.978]	0.352 [0.269-0.442]	1.663 [1.396-1.981]	0.222 [0.095-0.518]
PCT IP-10 PTX3	202	0.809 [0.741-0.876]	0.793	0.664 [0.583-0.770]	0.661	0.898	0.516 [0.434-0.598]	0.940 [0.867-0.980]	0.373 [0.286-0.467]	1.857 [1.537-2.243]	0.198 [0.085-0.460]
IL-6 PTX3 CD14	202	0.869 [0.807-0.931]	0.855	0.663 [0.593-0.774]	0.631	0.898	0.556 [0.473-0.636]	0.944 [0.875-0.982]	0.393 [0.302-0.490]	2.020 [1.653-2.470]	0.184 [0.079-0.427]
IP-10 NGAL gelsolin	199	0.856 [0.791-0.920]	0.851	0.663 [0.536-0.821]	0.616	0.898	0.660 [0.578-0.735]	0.952 [0.891-0.984]	0.463 [0.360-0.568]	2.641 [2.073-3.365]	0.155 [0.067-0.358]
IL-6 PCT CD14	202	0.873 [0.811-0.935]	0.862	0.663 [0.586-0.779]	0.628	0.898	0.536 [0.454-0.617]	0.943 [0.871-0.981]	0.383 [0.294-0.478]	1.935 [1.593-2.351]	0.190 [0.082-0.443]
IL-10 CD14 LBP	202	0.872 [0.810-0.934]	0.862	0.662 [0.613-0.775]	0.655	0.898	0.516 [0.434-0.598]	0.940 [0.867-0.980]	0.373 [0.286-0.467]	1.857 [1.537-2.243]	0.198 [0.085-0.460]
IL-6 IL-10 NGAL	202	0.890 [0.831-0.950]	0.882	0.661 [0.581-0.832]	0.626	0.898	0.641 [0.559-0.716]	0.951 [0.890-0.984]	0.444 [0.345-0.548]	2.498 [1.982-3.149]	0.159 [0.069-0.369]
IL-6 LBP NGAL	202	0.875 [0.812-0.937]	0.862	0.661 [0.568-0.805]	0.636	0.898	0.634 [0.552-0.710]	0.951 [0.889-0.984]	0.440 [0.341-0.543]	2.453 [1.951-3.084]	0.161 [0.070-0.373]
IL-6 PCT IP-10	204	0.876 [0.816-0.937]	0.873	0.661 [0.586-0.796]	0.62	0.902	0.529 [0.447-0.611]	0.942 [0.870-0.981]	0.390 [0.301-0.484]	1.917 [1.584-2.320]	0.185 [0.079-0.431]
PCT PTX3 calprotectin	199	0.773 [0.703-0.843]	0.756	0.659 [0.599-0.742]	0.644	0.898	0.467 [0.385-0.550]	0.933 [0.851-0.978]	0.355 [0.271-0.446]	1.684 [1.411-2.010]	0.219 [0.094-0.511]
IL-6 PCT LBP	202	0.873 [0.810-0.936]	0.866	0.659 [0.582-0.779]	0.622	0.898	0.477 [0.396-0.559]	0.936 [0.857-0.979]	0.355 [0.271-0.446]	1.717 [1.437-2.053]	0.214 [0.092-0.499]
IL-6 NGAL calprotectin	199	0.874 [0.811-0.937]	0.866	0.659 [0.565-0.800]	0.621	0.898	0.593 [0.510-0.673]	0.947 [0.880-0.983]	0.419 [0.323-0.519]	2.208 [1.781-2.738]	0.172 [0.074-0.399]
IL-6 IP-10 PTX3	202	0.869 [0.806-0.932]	0.858	0.658 [0.589-0.774]	0.626	0.898	0.549 [0.467-0.629]	0.944 [0.874-0.982]	0.389 [0.299-0.486]	1.991 [1.632-2.429]	0.186 [0.080-0.432]
IL-6 PTX3 LBP	202	0.870 [0.807-0.933]	0.858	0.656 [0.592-0.773]	0.621	0.898	0.556 [0.473-0.636]	0.944 [0.875-0.982]	0.393 [0.302-0.490]	2.020 [1.653-2.470]	0.184 [0.079-0.427]
PCT calprotectin gelsolin	199	0.748 [0.676-0.821]	0.727	0.655 [0.602-0.738]	0.647	0.898	0.507 [0.424-0.589]	0.938 [0.862-0.980]	0.373 [0.286-0.467]	1.820 [1.509-2.196]	0.201 [0.086-0.469]
PCT LBP NGAL	202	0.839 [0.773-0.905]	0.825	0.655 [0.563-0.796]	0.643	0.898	0.608 [0.526-0.686]	0.949 [0.885-0.983]	0.423 [0.327-0.524]	2.290 [1.840-2.850]	0.168 [0.072-0.389]
CD14 LBP NGAL	202	0.839 [0.772-0.905]	0.824	0.653 [0.562-0.794]	0.639	0.898	0.621 [0.539-0.698]	0.950 [0.887-0.984]	0.431 [0.334-0.533]	2.369 [1.894-2.963]	0.164 [0.071-0.381]
CD14 NGAL gelsolin	199	0.847 [0.783-0.911]	0.836	0.653 [0.560-0.804]	0.623	0.898	0.640 [0.558-0.717]	0.950 [0.888-0.984]	0.449 [0.348-0.553]	2.494 [1.975-3.150]	0.159 [0.069-0.369]
IL-6 IP-10 NGAL	202	0.873 [0.809-0.937]	0.864	0.651 [0.551-0.805]	0.616	0.898	0.627 [0.546-0.704]	0.950 [0.888-0.984]	0.436 [0.337-0.538]	2.410 [1.922-3.022]	0.163 [0.070-0.377]
IP-10 IL-10 CD14	202	0.865 [0.801-0.930]	0.858	0.647 [0.571-0.793]	0.611	0.898	0.601 [0.519-0.679]	0.948 [0.884-0.983]	0.419 [0.323-0.519]	2.252 [1.814-2.796]	0.170 [0.073-0.393]
IL-6 IP-10 IL-10	204	0.878 [0.816-0.940]	0.868	0.645 [0.568-0.797]	0.607	0.902	0.536 [0.454-0.617]	0.943 [0.871-0.981]	0.393 [0.304-0.488]	1.944 [1.603-2.357]	0.183 [0.079-0.426]
PCT PTX3 LBP	202	0.817 [0.749-0.885]	0.802	0.645 [0.583-0.763]	0.631	0.898	0.582 [0.499-0.661]	0.947 [0.880-0.983]	0.407 [0.314-0.506]	2.147 [1.741-2.647]	0.175 [0.076-0.407]
IL-10 CD14 calprotectin	199	0.861 [0.795-0.928]	0.853	0.644 [0.586-0.756]	0.612	0.898	0.480 [0.398-0.563]	0.935 [0.855-0.979]	0.361 [0.276-0.453]	1.727 [1.442-2.068]	0.213 [0.091-0.496]
IL-6 IL-10 LBP	202	0.880 [0.818-0.943]	0.874	0.643 [0.577-0.806]	0.608	0.898	0.549 [0.467-0.629]	0.944 [0.874-0.982]	0.389 [0.299-0.486]	1.991 [1.632-2.429]	0.186 [0.080-0.432]
LBP NGAL calprotectin	199	0.835 [0.767-0.903]	0.824	0.643 [0.539-0.794]	0.61	0.898	0.620 [0.537-0.698]	0.949 [0.885-0.983]	0.436 [0.337-0.538]	2.363 [1.887-2.960]	0.165 [0.071-0.381]
IL-6 calprotectin gelsolin	199	0.862 [0.796-0.927]	0.853	0.642 [0.587-0.762]	0.609	0.898	0.513 [0.430-0.596]	0.939 [0.863-0.980]	0.376 [0.288-0.470]	1.845 [1.527-2.230]	0.199 [0.085-0.463]
PTX3 CD14 LBP	202	0.816 [0.746-0.886]	0.805	0.642 [0.562-0.757]	0.614	0.898	0.536 [0.454-0.617]	0.943 [0.871-0.981]	0.383 [0.294-0.478]	1.935 [1.593-2.351]	0.190 [0.082-0.443]
IP-10 PTX3 calprotectin	199	0.800 [0.728-0.871]	0.784	0.640 [0.583-0.733]	0.628	0.898	0.447 [0.366-0.530]	0.931 [0.845-0.977]	0.346 [0.264-0.436]	1.623 [1.366-1.927]	0.228 [0.098-0.534]
PTX3 LBP calprotectin	199	0.815 [0.746-0.884]	0.803	0.638 [0.559-0.766]	0.612	0.898	0.593 [0.510-0.673]	0.947 [0.880-0.983]	0.419 [0.323-0.519]	2.208 [1.781-2.738]	0.172 [0.074-0.399]
IL-6 IL-10 CD14	202	0.881 [0.818-0.944]	0.872	0.638 [0.558-0.816]	0.59	0.898	0.549 [0.467-0.629]	0.944 [0.874-0.982]	0.389 [0.299-0.486]	1.991 [1.632-2.429]	0.186 [0.080-0.432]
IL-6 CD14 calprotectin	199	0.859 [0.791-0.926]	0.850	0.638 [0.562-0.757]	0.597	0.898	0.500 [0.417-0.583]	0.938 [0.860-0.979]	0.370 [0.283-0.463]	1.796 [1.491-2.163]	0.204 [0.088-0.476]
IL-6 IL-10 calprotectin	201	0.874 [0.811-0.937]	0.869	0.638 [0.573-0.781]	0.603	0.902	0.500 [0.417-0.583]	0.938 [0.860-0.979]	0.380 [0.293-0.473]	1.804 [1.501-2.168]	0.196 [0.084-0.458]
IL-6 CD14 gelsolin	199	0.859 [0.792-0.926]	0.851	0.637 [0.566-0.754]	0.583	0.898	0.433 [0.353-0.517]	0.929 [0.841-0.976]	0.341 [0.260-0.430]	1.585 [1.339-1.876]	0.235 [0.101-0.551]
PCT IP-10 calprotectin	201	0.801 [0.732-0.870]	0.784	0.636 [0.553-0.765]	0.607	0.902	0.500 [0.417-0.583]	0.938 [0.860-0.979]	0.380 [0.293-0.473]	1.804 [1.501-2.168]	0.196 [0.084-0.458]

IL-6 LBP calprotectin	199	0.859 [0.791-0.927]	0.853	0.636 [0.558-0.751]	0.574	0.898	0.453 [0.372-0.537]	0.932 [0.847-0.977]	0.349 [0.266-0.439]	1.643 [1.381-1.954]	0.225 [0.096-0.526]
PTX3 LBP gelsolin	199	0.811 [0.741-0.881]	0.792	0.635 [0.559-0.747]	0.606	0.898	0.513 [0.430-0.596]	0.939 [0.863-0.980]	0.376 [0.288-0.470]	1.845 [1.527-2.230]	0.199 [0.085-0.463]
PTX3 calprotectin gelsolin	199	0.759 [0.685-0.832]	0.740	0.634 [0.562-0.726]	0.605	0.898	0.453 [0.372-0.537]	0.932 [0.847-0.977]	0.349 [0.266-0.439]	1.643 [1.381-1.954]	0.225 [0.096-0.526]
PCT IP-10 NGAL	202	0.850 [0.784-0.917]	0.840	0.633 [0.516-0.805]	0.565	0.898	0.641 [0.559-0.716]	0.951 [0.890-0.984]	0.444 [0.345-0.548]	2.498 [1.982-3.149]	0.159 [0.069-0.369]
PCT NGAL calprotectin	199	0.826 [0.757-0.896]	0.816	0.632 [0.526-0.795]	0.589	0.898	0.620 [0.537-0.698]	0.949 [0.885-0.983]	0.436 [0.337-0.538]	2.363 [1.887-2.960]	0.165 [0.071-0.381]
IP-10 calprotectin gelsolin	199	0.778 [0.702-0.853]	0.756	0.630 [0.556-0.723]	0.614	0.898	0.440 [0.359-0.523]	0.930 [0.843-0.977]	0.344 [0.262-0.433]	1.603 [1.352-1.901]	0.232 [0.099-0.543]
IP-10 LBP NGAL	202	0.852 [0.784-0.920]	0.843	0.630 [0.523-0.789]	0.535	0.898	0.575 [0.493-0.655]	0.946 [0.879-0.982]	0.404 [0.311-0.502]	2.114 [1.718-2.600]	0.177 [0.076-0.412]
IL-6 LBP gelsolin	199	0.863 [0.795-0.930]	0.857	0.626 [0.554-0.760]	0.558	0.898	0.480 [0.398-0.563]	0.935 [0.855-0.979]	0.361 [0.276-0.453]	1.727 [1.442-2.068]	0.213 [0.091-0.496]
IL-6 CD14 LBP	202	0.860 [0.792-0.929]	0.854	0.624 [0.548-0.754]	0.555	0.898	0.431 [0.352-0.514]	0.930 [0.843-0.977]	0.336 [0.256-0.424]	1.579 [1.336-1.867]	0.237 [0.101-0.554]
PCT PTX3 gelsolin	199	0.756 [0.681-0.831]	0.733	0.624 [0.563-0.729]	0.61	0.898	0.493 [0.411-0.576]	0.937 [0.858-0.979]	0.367 [0.281-0.459]	1.772 [1.474-2.130]	0.207 [0.089-0.482]
PCT LBP calprotectin	199	0.805 [0.731-0.878]	0.785	0.623 [0.540-0.728]	0.587	0.898	0.453 [0.372-0.537]	0.932 [0.847-0.977]	0.349 [0.266-0.439]	1.643 [1.381-1.954]	0.225 [0.096-0.526]
IP-10 PTX3 CD14	202	0.811 [0.738-0.883]	0.794	0.623 [0.541-0.734]	0.577	0.898	0.503 [0.421-0.585]	0.939 [0.863-0.980]	0.367 [0.281-0.459]	1.808 [1.502-2.176]	0.203 [0.087-0.472]
PCT PTX3 CD14	202	0.804 [0.733-0.876]	0.782	0.621 [0.536-0.746]	0.559	0.898	0.392 [0.314-0.474]	0.923 [0.830-0.975]	0.321 [0.244-0.406]	1.477 [1.261-1.731]	0.260 [0.111-0.611]
IL-6 IP-10 gelsolin	199	0.861 [0.793-0.929]	0.853	0.621 [0.560-0.769]	0.564	0.898	0.407 [0.327-0.490]	0.924 [0.832-0.975]	0.331 [0.252-0.418]	1.513 [1.286-1.781]	0.251 [0.107-0.589]
PTX3 CD14 gelsolin	199	0.790 [0.716-0.864]	0.766	0.621 [0.549-0.715]	0.581	0.898	0.460 [0.378-0.543]	0.932 [0.849-0.978]	0.352 [0.269-0.442]	1.663 [1.396-1.981]	0.222 [0.095-0.518]
PCT CD14 NGAL	202	0.833 [0.763-0.902]	0.820	0.619 [0.529-0.780]	0.577	0.898	0.601 [0.519-0.679]	0.948 [0.884-0.983]	0.419 [0.323-0.519]	2.252 [1.814-2.796]	0.170 [0.073-0.393]
IL-6 IP-10 calprotectin	201	0.859 [0.792-0.926]	0.851	0.619 [0.558-0.759]	0.572	0.902	0.413 [0.334-0.497]	0.925 [0.834-0.975]	0.343 [0.263-0.430]	1.537 [1.308-1.808]	0.237 [0.101-0.557]
PTX3 CD14 calprotectin	199	0.790 [0.717-0.863]	0.772	0.619 [0.562-0.720]	0.588	0.898	0.420 [0.340-0.503]	0.926 [0.837-0.976]	0.336 [0.256-0.424]	1.548 [1.312-1.827]	0.243 [0.104-0.569]
IP-10 IL-10 calprotectin	201	0.845 [0.775-0.914]	0.837	0.616 [0.535-0.734]	0.565	0.902	0.480 [0.398-0.563]	0.935 [0.855-0.979]	0.371 [0.286-0.462]	1.735 [1.451-2.073]	0.204 [0.087-0.477]
IP-10 NGAL calprotectin	199	0.844 [0.775-0.913]	0.834	0.614 [0.511-0.796]	0.507	0.898	0.640 [0.558-0.717]	0.950 [0.888-0.984]	0.449 [0.348-0.553]	2.494 [1.975-3.150]	0.159 [0.069-0.369]
CD14 LBP calprotectin	199	0.801 [0.727-0.874]	0.785	0.612 [0.536-0.735]	0.54	0.898	0.480 [0.398-0.563]	0.935 [0.855-0.979]	0.361 [0.276-0.453]	1.727 [1.442-2.068]	0.213 [0.091-0.496]
IL-6 IP-10 LBP	202	0.861 [0.791-0.931]	0.855	0.611 [0.540-0.759]	0.538	0.898	0.425 [0.345-0.507]	0.929 [0.841-0.976]	0.333 [0.254-0.421]	1.561 [1.323-1.843]	0.240 [0.103-0.562]
CD14 NGAL calprotectin	199	0.831 [0.760-0.901]	0.817	0.611 [0.521-0.783]	0.554	0.898	0.627 [0.544-0.704]	0.949 [0.886-0.983]	0.440 [0.341-0.543]	2.405 [1.915-3.021]	0.163 [0.070-0.377]
PCT LBP gelsolin	199	0.801 [0.727-0.875]	0.779	0.611 [0.511-0.739]	0.536	0.898	0.533 [0.450-0.615]	0.941 [0.868-0.981]	0.386 [0.296-0.482]	1.924 [1.583-2.339]	0.191 [0.082-0.445]
IL-6 IP-10 CD14	202	0.857 [0.787-0.927]	0.846	0.610 [0.543-0.753]	0.554	0.898	0.477 [0.396-0.559]	0.936 [0.857-0.979]	0.355 [0.271-0.446]	1.717 [1.437-2.053]	0.214 [0.092-0.499]
IP-10 PTX3 LBP	202	0.832 [0.760-0.903]	0.824	0.609 [0.534-0.742]	0.561	0.898	0.438 [0.358-0.520]	0.931 [0.845-0.977]	0.338 [0.258-0.427]	1.598 [1.349-1.891]	0.233 [0.100-0.545]
IP-10 CD14 NGAL	202	0.847 [0.777-0.916]	0.838	0.608 [0.508-0.791]	0.518	0.898	0.614 [0.532-0.692]	0.949 [0.886-0.983]	0.427 [0.330-0.528]	2.329 [1.867-2.905]	0.166 [0.072-0.385]
IP-10 PTX3 gelsolin	199	0.784 [0.706-0.862]	0.771	0.607 [0.530-0.698]	0.573	0.898	0.393 [0.315-0.476]	0.922 [0.827-0.974]	0.326 [0.248-0.412]	1.480 [1.262-1.737]	0.259 [0.110-0.609]
LBP calprotectin gelsolin	199	0.800 [0.726-0.874]	0.788	0.604 [0.536-0.733]	0.535	0.898	0.473 [0.391-0.556]	0.934 [0.853-0.978]	0.358 [0.273-0.449]	1.705 [1.426-2.039]	0.216 [0.092-0.503]
IP-10 LBP calprotectin	199	0.822 [0.746-0.899]	0.818	0.593 [0.505-0.724]	0.519	0.898	0.433 [0.353-0.517]	0.929 [0.841-0.976]	0.341 [0.260-0.430]	1.585 [1.339-1.876]	0.235 [0.101-0.551]
PCT CD14 LBP	202	0.802 [0.725-0.879]	0.782	0.587 [0.492-0.737]	0.498	0.898	0.536 [0.454-0.617]	0.943 [0.871-0.981]	0.383 [0.294-0.478]	1.935 [1.593-2.351]	0.190 [0.082-0.443]
PCT IP-10 LBP	202	0.827 [0.752-0.902]	0.814	0.578 [0.503-0.746]	0.494	0.898	0.556 [0.473-0.636]	0.944 [0.875-0.982]	0.393 [0.302-0.490]	2.020 [1.653-2.470]	0.184 [0.079-0.427]
PCT IP-10 gelsolin	199	0.782 [0.703-0.861]	0.765	0.576 [0.515-0.705]	0.534	0.898	0.413 [0.334-0.497]	0.925 [0.834-0.975]	0.333 [0.254-0.421]	1.531 [1.299-1.804]	0.247 [0.105-0.579]
CD14 LBP gelsolin	199	0.790 [0.712-0.868]	0.771	0.576 [0.513-0.711]	0.503	0.898	0.460 [0.378-0.543]	0.932 [0.849-0.978]	0.352 [0.269-0.442]	1.663 [1.396-1.981]	0.222 [0.095-0.518]

PCT CD14 calprotectin	199	0.779 [0.700-0.858]	0.762	0.572 [0.529-0.698]	0.507	0.898	0.260 [0.192-0.338]	0.886 [0.754-0.962]	0.284 [0.214-0.362]	1.213 [1.061-1.387]	0.392 [0.164-0.940]
CD14 calprotectin gelsolin	199	0.762 [0.682-0.842]	0.753	0.570 [0.532-0.693]	0.528	0.898	0.267 [0.198-0.345]	0.889 [0.759-0.963]	0.286 [0.216-0.364]	1.224 [1.070-1.401]	0.383 [0.160-0.915]
IP-10 CD14 calprotectin	199	0.802 [0.724-0.881]	0.789	0.564 [0.497-0.716]	0.478	0.898	0.507 [0.424-0.589]	0.938 [0.862-0.980]	0.373 [0.286-0.467]	1.820 [1.509-2.196]	0.201 [0.086-0.469]
IP-10 CD14 LBP	202	0.817 [0.739-0.896]	0.804	0.564 [0.484-0.717]	0.438	0.898	0.444 [0.364-0.527]	0.932 [0.847-0.977]	0.341 [0.260-0.430]	1.616 [1.363-1.916]	0.230 [0.098-0.537]
IP-10 LBP gelsolin	199	0.811 [0.732-0.891]	0.803	0.562 [0.501-0.698]	0.486	0.898	0.387 [0.308-0.470]	0.921 [0.824-0.974]	0.324 [0.246-0.409]	1.464 [1.250-1.715]	0.264 [0.112-0.620]
PCT IP-10 CD14	202	0.805 [0.726-0.884]	0.796	0.558 [0.500-0.712]	0.482	0.898	0.386 [0.308-0.468]	0.922 [0.827-0.974]	0.319 [0.242-0.404]	1.462 [1.249-1.710]	0.265 [0.113-0.622]
IP-10 CD14 gelsolin	199	0.784 [0.701-0.868]	0.772	0.552 [0.486-0.680]	0.472	0.898	0.360 [0.283-0.442]	0.915 [0.813-0.972]	0.314 [0.239-0.398]	1.403 [1.204-1.635]	0.283 [0.120-0.668]
PCT CD14 gelsolin	199	0.774 [0.693-0.855]	0.753	0.549 [0.487-0.700]	0.438	0.898	0.353 [0.277-0.435]	0.914 [0.810-0.971]	0.312 [0.237-0.395]	1.389 [1.194-1.615]	0.289 [0.122-0.681]
PCT PTX3 NGAL gelsolin	199	0.863 [0.810-0.917]	0.842	0.760 [0.657-0.856]	0.76	0.898	0.700 [0.620-0.772]	0.955 [0.897-0.985]	0.494 [0.387-0.602]	2.993 [2.303-3.890]	0.146 [0.063-0.337]
PTX3 NGAL calprotectin gelsolin	199	0.861 [0.806-0.916]	0.841	0.756 [0.634-0.863]	0.766	0.898	0.727 [0.648-0.796]	0.956 [0.901-0.986]	0.518 [0.407-0.627]	3.285 [2.489-4.336]	0.140 [0.061-0.324]
PCT IL-10 NGAL gelsolin	199	0.905 [0.857-0.953]	0.889	0.743 [0.665-0.860]	0.729	0.898	0.747 [0.669-0.814]	0.957 [0.903-0.986]	0.537 [0.423-0.647]	3.545 [2.651-4.739]	0.137 [0.059-0.315]
PCT IL-10 LBP gelsolin	199	0.893 [0.842-0.945]	0.876	0.740 [0.683-0.820]	0.729	0.898	0.607 [0.524-0.685]	0.948 [0.883-0.983]	0.427 [0.330-0.528]	2.283 [1.832-2.845]	0.168 [0.073-0.390]
IL-10 PTX3 NGAL gelsolin	199	0.893 [0.842-0.945]	0.883	0.731 [0.645-0.842]	0.726	0.898	0.693 [0.613-0.766]	0.954 [0.896-0.985]	0.489 [0.382-0.597]	2.928 [2.261-3.792]	0.147 [0.064-0.340]
IL-6 PCT NGAL gelsolin	199	0.892 [0.840-0.944]	0.879	0.730 [0.661-0.822]	0.719	0.898	0.607 [0.524-0.685]	0.948 [0.883-0.983]	0.427 [0.330-0.528]	2.283 [1.832-2.845]	0.168 [0.073-0.390]
IL-10 CD14 NGAL gelsolin	199	0.894 [0.841-0.946]	0.880	0.728 [0.624-0.843]	0.716	0.898	0.687 [0.606-0.760]	0.954 [0.895-0.985]	0.484 [0.377-0.591]	2.866 [2.221-3.698]	0.149 [0.064-0.343]
IL-10 NGAL calprotectin gelsolin	199	0.895 [0.843-0.947]	0.883	0.727 [0.633-0.842]	0.717	0.898	0.687 [0.606-0.760]	0.954 [0.895-0.985]	0.484 [0.377-0.591]	2.866 [2.221-3.698]	0.149 [0.064-0.343]
PTX3 LBP NGAL gelsolin	199	0.863 [0.807-0.919]	0.841	0.724 [0.636-0.830]	0.728	0.898	0.660 [0.578-0.735]	0.952 [0.891-0.984]	0.463 [0.360-0.568]	2.641 [2.073-3.365]	0.155 [0.067-0.358]
IP-10 IL-10 NGAL gelsolin	199	0.895 [0.842-0.947]	0.886	0.724 [0.626-0.847]	0.709	0.898	0.733 [0.655-0.802]	0.957 [0.901-0.986]	0.524 [0.412-0.634]	3.367 [2.541-4.463]	0.139 [0.060-0.321]
IL-10 LBP NGAL gelsolin	199	0.895 [0.843-0.947]	0.883	0.721 [0.650-0.830]	0.712	0.898	0.620 [0.537-0.698]	0.949 [0.885-0.983]	0.436 [0.337-0.538]	2.363 [1.887-2.960]	0.165 [0.071-0.381]
PTX3 CD14 NGAL gelsolin	199	0.857 [0.800-0.914]	0.832	0.721 [0.615-0.839]	0.721	0.898	0.693 [0.613-0.766]	0.954 [0.896-0.985]	0.489 [0.382-0.597]	2.928 [2.261-3.792]	0.147 [0.064-0.340]
IP-10 PTX3 NGAL gelsolin	199	0.863 [0.804-0.922]	0.844	0.721 [0.571-0.848]	0.702	0.898	0.680 [0.599-0.754]	0.953 [0.894-0.985]	0.478 [0.373-0.585]	2.806 [2.182-3.609]	0.150 [0.065-0.347]
PCT IL-10 PTX3 LBP	202	0.887 [0.831-0.942]	0.871	0.720 [0.659-0.798]	0.716	0.898	0.569 [0.486-0.648]	0.946 [0.878-0.982]	0.400 [0.308-0.498]	2.082 [1.696-2.555]	0.179 [0.077-0.417]
PCT IP-10 NGAL gelsolin	199	0.868 [0.810-0.925]	0.850	0.718 [0.584-0.846]	0.709	0.898	0.687 [0.606-0.760]	0.954 [0.895-0.985]	0.484 [0.377-0.591]	2.866 [2.221-3.698]	0.149 [0.064-0.343]
PCT PTX3 NGAL calprotectin	199	0.847 [0.787-0.907]	0.819	0.716 [0.586-0.840]	0.71	0.898	0.700 [0.620-0.772]	0.955 [0.897-0.985]	0.494 [0.387-0.602]	2.993 [2.303-3.890]	0.146 [0.063-0.337]
IL-10 PTX3 CD14 NGAL	202	0.886 [0.831-0.942]	0.873	0.715 [0.617-0.830]	0.707	0.898	0.647 [0.566-0.723]	0.952 [0.891-0.984]	0.449 [0.348-0.553]	2.544 [2.013-3.216]	0.158 [0.068-0.365]
PCT LBP NGAL gelsolin	199	0.856 [0.799-0.913]	0.836	0.714 [0.645-0.812]	0.709	0.898	0.613 [0.530-0.692]	0.948 [0.884-0.983]	0.431 [0.334-0.533]	2.322 [1.859-2.901]	0.166 [0.072-0.385]
PCT IL-10 PTX3 NGAL	202	0.888 [0.833-0.944]	0.876	0.713 [0.615-0.829]	0.706	0.898	0.673 [0.593-0.747]	0.954 [0.895-0.985]	0.468 [0.364-0.574]	2.748 [2.148-3.515]	0.152 [0.066-0.350]
IL-10 PTX3 LBP gelsolin	199	0.883 [0.826-0.939]	0.865	0.712 [0.648-0.798]	0.698	0.898	0.567 [0.483-0.647]	0.944 [0.875-0.982]	0.404 [0.311-0.502]	2.072 [1.687-2.546]	0.180 [0.078-0.418]
IL-6 PCT PTX3 gelsolin	199	0.884 [0.828-0.939]	0.870	0.712 [0.658-0.800]	0.695	0.898	0.607 [0.524-0.685]	0.948 [0.883-0.983]	0.427 [0.330-0.528]	2.283 [1.832-2.845]	0.168 [0.073-0.390]
PCT IL-10 PTX3 gelsolin	199	0.887 [0.833-0.942]	0.872	0.710 [0.643-0.824]	0.687	0.898	0.660 [0.578-0.735]	0.952 [0.891-0.984]	0.463 [0.360-0.568]	2.641 [2.073-3.365]	0.155 [0.067-0.358]
IP-10 IL-10 PTX3 NGAL	202	0.887 [0.831-0.943]	0.870	0.709 [0.618-0.825]	0.698	0.898	0.667 [0.586-0.741]	0.953 [0.894-0.985]	0.463 [0.360-0.568]	2.694 [2.112-3.435]	0.153 [0.066-0.354]
PCT IL-10 calprotectin gelsolin	199	0.891 [0.837-0.946]	0.877	0.707 [0.629-0.835]	0.683	0.898	0.673 [0.592-0.748]	0.953 [0.893-0.985]	0.473 [0.369-0.579]	2.749 [2.144-3.524]	0.152 [0.066-0.350]
IL-6 IL-10 NGAL gelsolin	199	0.898 [0.845-0.951]	0.880	0.707 [0.638-0.843]	0.683	0.898	0.593 [0.510-0.673]	0.947 [0.880-0.983]	0.419 [0.323-0.519]	2.208 [1.781-2.738]	0.172 [0.074-0.399]

IL-6 PCT IL-10 PTX3	202	0.895 [0.841-0.950]	0.883	0.707 [0.623-0.827]	0.685	0.898	0.608 [0.526-0.686]	0.949 [0.885-0.983]	0.423 [0.327-0.524]	2.290 [1.840-2.850]	0.168 [0.072-0.389]
IL-6 PTX3 NGAL gelsolin	199	0.885 [0.830-0.940]	0.867	0.707 [0.647-0.813]	0.704	0.898	0.580 [0.497-0.660]	0.946 [0.878-0.982]	0.411 [0.317-0.510]	2.138 [1.732-2.639]	0.176 [0.076-0.408]
PCT IP-10 calprotectin gelsolin	199	0.822 [0.759-0.884]	0.800	0.706 [0.632-0.796]	0.703	0.898	0.620 [0.537-0.698]	0.949 [0.885-0.983]	0.436 [0.337-0.538]	2.363 [1.887-2.960]	0.165 [0.071-0.381]
PCT IL-10 CD14 LBP	202	0.884 [0.828-0.940]	0.868	0.705 [0.656-0.798]	0.71	0.898	0.601 [0.519-0.679]	0.948 [0.884-0.983]	0.419 [0.323-0.519]	2.252 [1.814-2.796]	0.170 [0.073-0.393]
PCT IP-10 IL-10 LBP	202	0.884 [0.828-0.940]	0.869	0.704 [0.650-0.801]	0.703	0.898	0.556 [0.473-0.636]	0.944 [0.875-0.982]	0.393 [0.302-0.490]	2.020 [1.653-2.470]	0.184 [0.079-0.427]
IL-10 PTX3 CD14 gelsolin	199	0.876 [0.817-0.934]	0.857	0.703 [0.600-0.806]	0.693	0.898	0.620 [0.537-0.698]	0.949 [0.885-0.983]	0.436 [0.337-0.538]	2.363 [1.887-2.960]	0.165 [0.071-0.381]
PCT IP-10 IL-10 PTX3	202	0.880 [0.823-0.937]	0.867	0.703 [0.640-0.797]	0.687	0.898	0.608 [0.526-0.686]	0.949 [0.885-0.983]	0.423 [0.327-0.524]	2.290 [1.840-2.850]	0.168 [0.072-0.389]
PTX3 LBP NGAL calprotectin	199	0.852 [0.791-0.913]	0.829	0.702 [0.567-0.824]	0.689	0.898	0.640 [0.558-0.717]	0.950 [0.888-0.984]	0.449 [0.348-0.553]	2.494 [1.975-3.150]	0.159 [0.069-0.369]
IL-10 PTX3 NGAL calprotectin	199	0.884 [0.827-0.941]	0.870	0.702 [0.610-0.817]	0.693	0.898	0.640 [0.558-0.717]	0.950 [0.888-0.984]	0.449 [0.348-0.553]	2.494 [1.975-3.150]	0.159 [0.069-0.369]
IL-10 LBP calprotectin gelsolin	199	0.880 [0.822-0.939]	0.867	0.702 [0.653-0.781]	0.689	0.898	0.493 [0.411-0.576]	0.937 [0.858-0.979]	0.367 [0.281-0.459]	1.772 [1.474-2.130]	0.207 [0.089-0.482]
IL-6 PCT IL-10 gelsolin	199	0.897 [0.843-0.951]	0.882	0.702 [0.628-0.831]	0.681	0.898	0.687 [0.606-0.760]	0.954 [0.895-0.985]	0.484 [0.377-0.591]	2.866 [2.221-3.698]	0.149 [0.064-0.343]
IL-6 PCT calprotectin gelsolin	199	0.880 [0.823-0.937]	0.867	0.700 [0.652-0.801]	0.679	0.898	0.500 [0.417-0.583]	0.938 [0.860-0.979]	0.370 [0.283-0.463]	1.796 [1.491-2.163]	0.204 [0.088-0.476]
PCT IL-10 CD14 NGAL	202	0.888 [0.831-0.945]	0.875	0.700 [0.580-0.840]	0.678	0.898	0.680 [0.600-0.753]	0.954 [0.896-0.985]	0.473 [0.369-0.579]	2.804 [2.185-3.598]	0.150 [0.065-0.347]
PCT PTX3 LBP NGAL	202	0.851 [0.791-0.911]	0.827	0.700 [0.601-0.823]	0.699	0.898	0.660 [0.579-0.735]	0.953 [0.893-0.985]	0.458 [0.356-0.563]	2.642 [2.078-3.359]	0.155 [0.067-0.357]
IP-10 IL-10 PTX3 gelsolin	199	0.870 [0.808-0.932]	0.851	0.699 [0.602-0.787]	0.68	0.898	0.527 [0.444-0.609]	0.940 [0.867-0.980]	0.383 [0.294-0.478]	1.897 [1.563-2.302]	0.194 [0.083-0.451]
PCT NGAL calprotectin gelsolin	199	0.849 [0.788-0.909]	0.832	0.699 [0.596-0.822]	0.689	0.898	0.633 [0.551-0.710]	0.950 [0.887-0.984]	0.444 [0.345-0.548]	2.449 [1.945-3.084]	0.161 [0.070-0.373]
IL-6 PCT PTX3 calprotectin	199	0.880 [0.823-0.938]	0.868	0.698 [0.635-0.792]	0.676	0.898	0.587 [0.503-0.666]	0.946 [0.879-0.982]	0.415 [0.320-0.515]	2.172 [1.756-2.687]	0.174 [0.075-0.403]
PTX3 CD14 NGAL calprotectin	199	0.847 [0.786-0.909]	0.822	0.698 [0.570-0.829]	0.685	0.898	0.673 [0.592-0.748]	0.953 [0.893-0.985]	0.473 [0.369-0.579]	2.749 [2.144-3.524]	0.152 [0.066-0.350]
IL-6 CD14 NGAL gelsolin	199	0.880 [0.823-0.937]	0.867	0.697 [0.624-0.805]	0.687	0.898	0.580 [0.497-0.660]	0.946 [0.878-0.982]	0.411 [0.317-0.510]	2.138 [1.732-2.639]	0.176 [0.076-0.408]
PCT IP-10 PTX3 NGAL	202	0.860 [0.799-0.921]	0.842	0.696 [0.550-0.850]	0.651	0.898	0.706 [0.627-0.777]	0.956 [0.900-0.985]	0.494 [0.387-0.602]	3.053 [2.347-3.971]	0.145 [0.063-0.334]
IL-10 CD14 LBP gelsolin	199	0.878 [0.819-0.936]	0.863	0.696 [0.629-0.787]	0.687	0.898	0.553 [0.470-0.634]	0.943 [0.872-0.981]	0.396 [0.305-0.494]	2.010 [1.643-2.459]	0.184 [0.079-0.428]
IL-6 PCT PTX3 NGAL	202	0.886 [0.831-0.942]	0.870	0.695 [0.627-0.823]	0.679	0.898	0.647 [0.566-0.723]	0.952 [0.891-0.984]	0.449 [0.348-0.553]	2.544 [2.013-3.216]	0.158 [0.068-0.365]
IL-6 LBP NGAL gelsolin	199	0.881 [0.824-0.939]	0.863	0.695 [0.624-0.802]	0.689	0.898	0.573 [0.490-0.654]	0.945 [0.876-0.982]	0.407 [0.314-0.506]	2.105 [1.709-2.592]	0.178 [0.077-0.413]
PCT IP-10 IL-10 NGAL	202	0.887 [0.830-0.945]	0.870	0.694 [0.583-0.838]	0.674	0.898	0.673 [0.593-0.747]	0.954 [0.895-0.985]	0.468 [0.364-0.574]	2.748 [2.148-3.515]	0.152 [0.066-0.350]
IP-10 PTX3 NGAL calprotectin	199	0.858 [0.797-0.919]	0.838	0.693 [0.548-0.838]	0.638	0.898	0.687 [0.606-0.760]	0.954 [0.895-0.985]	0.484 [0.377-0.591]	2.866 [2.221-3.698]	0.149 [0.064-0.343]
IP-10 IL-10 LBP gelsolin	199	0.878 [0.820-0.937]	0.863	0.693 [0.629-0.788]	0.68	0.898	0.580 [0.497-0.660]	0.946 [0.878-0.982]	0.411 [0.317-0.510]	2.138 [1.732-2.639]	0.176 [0.076-0.408]
IL-6 IL-10 PTX3 gelsolin	199	0.885 [0.827-0.944]	0.873	0.693 [0.596-0.810]	0.671	0.898	0.520 [0.437-0.602]	0.940 [0.865-0.980]	0.379 [0.291-0.474]	1.871 [1.545-2.265]	0.196 [0.084-0.457]
IL-10 PTX3 LBP NGAL	202	0.885 [0.828-0.943]	0.873	0.693 [0.618-0.804]	0.699	0.898	0.601 [0.519-0.679]	0.948 [0.884-0.983]	0.419 [0.323-0.519]	2.252 [1.814-2.796]	0.170 [0.073-0.393]
IP-10 IL-10 PTX3 LBP	202	0.878 [0.819-0.937]	0.868	0.692 [0.636-0.783]	0.686	0.898	0.569 [0.486-0.648]	0.946 [0.878-0.982]	0.400 [0.308-0.498]	2.082 [1.696-2.555]	0.179 [0.077-0.417]
IL-6 NGAL calprotectin gelsolin	199	0.880 [0.822-0.938]	0.867	0.691 [0.619-0.800]	0.676	0.898	0.580 [0.497-0.660]	0.946 [0.878-0.982]	0.411 [0.317-0.510]	2.138 [1.732-2.639]	0.176 [0.076-0.408]
IL-6 PCT CD14 gelsolin	199	0.879 [0.820-0.937]	0.864	0.691 [0.643-0.786]	0.675	0.898	0.527 [0.444-0.609]	0.940 [0.867-0.980]	0.383 [0.294-0.478]	1.897 [1.563-2.302]	0.194 [0.083-0.451]
PTX3 CD14 LBP NGAL	202	0.851 [0.791-0.912]	0.828	0.691 [0.593-0.825]	0.691	0.898	0.634 [0.552-0.710]	0.951 [0.889-0.984]	0.440 [0.341-0.543]	2.453 [1.951-3.084]	0.161 [0.070-0.373]
IL-10 CD14 LBP NGAL	202	0.887 [0.829-0.944]	0.872	0.691 [0.625-0.803]	0.692	0.898	0.536 [0.454-0.617]	0.943 [0.871-0.981]	0.383 [0.294-0.478]	1.935 [1.593-2.351]	0.190 [0.082-0.443]
IP-10 IL-10 CD14 NGAL	202	0.882 [0.823-0.942]	0.868	0.690 [0.571-0.827]	0.67	0.898	0.647 [0.566-0.723]	0.952 [0.891-0.984]	0.449 [0.348-0.553]	2.544 [2.013-3.216]	0.158 [0.068-0.365]

PCT IL-10 CD14 gelsolin	199	0.889 [0.833-0.945]	0.875	0.690 [0.626-0.823]	0.668	0.898	0.620 [0.537-0.698]	0.949 [0.885-0.983]	0.436 [0.337-0.538]	2.363 [1.887-2.960]	0.165 [0.071-0.381]
IL-6 PCT PTX3 CD14	202	0.880 [0.822-0.938]	0.872	0.690 [0.622-0.795]	0.667	0.898	0.601 [0.519-0.679]	0.948 [0.884-0.983]	0.419 [0.323-0.519]	2.252 [1.814-2.796]	0.170 [0.073-0.393]
PCT IP-10 PTX3 calprotectin	199	0.822 [0.759-0.885]	0.796	0.690 [0.609-0.790]	0.686	0.898	0.553 [0.470-0.634]	0.943 [0.872-0.981]	0.396 [0.305-0.494]	2.010 [1.643-2.459]	0.184 [0.079-0.428]
IL-6 PCT PTX3 LBP	202	0.881 [0.823-0.938]	0.866	0.689 [0.621-0.798]	0.668	0.898	0.601 [0.519-0.679]	0.948 [0.884-0.983]	0.419 [0.323-0.519]	2.252 [1.814-2.796]	0.170 [0.073-0.393]
PCT IL-10 LBP calprotectin	199	0.881 [0.823-0.940]	0.868	0.688 [0.642-0.787]	0.663	0.898	0.533 [0.450-0.615]	0.941 [0.868-0.981]	0.386 [0.296-0.482]	1.924 [1.583-2.339]	0.191 [0.082-0.445]
PCT IP-10 IL-10 gelsolin	199	0.883 [0.826-0.940]	0.867	0.688 [0.624-0.824]	0.665	0.898	0.693 [0.613-0.766]	0.954 [0.896-0.985]	0.489 [0.382-0.597]	2.928 [2.261-3.792]	0.147 [0.064-0.340]
IL-10 PTX3 calprotectin gelsolin	199	0.876 [0.817-0.935]	0.860	0.688 [0.625-0.792]	0.676	0.898	0.573 [0.490-0.654]	0.945 [0.876-0.982]	0.407 [0.314-0.506]	2.105 [1.709-2.592]	0.178 [0.077-0.413]
IL-6 PCT IP-10 PTX3	202	0.881 [0.822-0.939]	0.870	0.688 [0.625-0.796]	0.662	0.898	0.582 [0.499-0.661]	0.947 [0.880-0.983]	0.407 [0.314-0.506]	2.147 [1.741-2.647]	0.175 [0.076-0.407]
PCT IL-10 PTX3 CD14	202	0.879 [0.822-0.937]	0.867	0.688 [0.639-0.802]	0.671	0.898	0.660 [0.579-0.735]	0.953 [0.893-0.985]	0.458 [0.356-0.563]	2.642 [2.078-3.359]	0.155 [0.067-0.357]
PCT IL-10 LBP NGAL	202	0.890 [0.833-0.947]	0.877	0.687 [0.601-0.824]	0.681	0.898	0.621 [0.539-0.698]	0.950 [0.887-0.984]	0.431 [0.334-0.533]	2.369 [1.894-2.963]	0.164 [0.071-0.381]
PCT IL-10 NGAL calprotectin	199	0.885 [0.827-0.943]	0.874	0.687 [0.571-0.830]	0.659	0.898	0.660 [0.578-0.735]	0.952 [0.891-0.984]	0.463 [0.360-0.568]	2.641 [2.073-3.365]	0.155 [0.067-0.358]
IL-6 PTX3 CD14 NGAL	202	0.882 [0.825-0.939]	0.868	0.686 [0.619-0.821]	0.673	0.898	0.673 [0.593-0.747]	0.954 [0.895-0.985]	0.468 [0.364-0.574]	2.748 [2.148-3.515]	0.152 [0.066-0.350]
IL-6 IP-10 IL-10 PTX3	202	0.883 [0.824-0.942]	0.871	0.686 [0.602-0.800]	0.671	0.898	0.536 [0.454-0.617]	0.943 [0.871-0.981]	0.383 [0.294-0.478]	1.935 [1.593-2.351]	0.190 [0.082-0.443]
CD14 LBP NGAL gelsolin	199	0.850 [0.788-0.911]	0.824	0.685 [0.601-0.805]	0.676	0.898	0.640 [0.558-0.717]	0.950 [0.888-0.984]	0.449 [0.348-0.553]	2.494 [1.975-3.150]	0.159 [0.069-0.369]
IL-6 IP-10 NGAL gelsolin	199	0.881 [0.823-0.939]	0.868	0.685 [0.614-0.804]	0.669	0.898	0.627 [0.544-0.704]	0.949 [0.886-0.983]	0.440 [0.341-0.543]	2.405 [1.915-3.021]	0.163 [0.070-0.377]
IL-6 PCT IP-10 IL-10	204	0.895 [0.839-0.950]	0.889	0.684 [0.616-0.820]	0.652	0.902	0.549 [0.467-0.629]	0.944 [0.874-0.982]	0.400 [0.310-0.496]	2.000 [1.643-2.435]	0.179 [0.077-0.416]
IL-10 CD14 calprotectin gelsolin	199	0.876 [0.816-0.936]	0.858	0.684 [0.606-0.804]	0.662	0.898	0.580 [0.497-0.660]	0.946 [0.878-0.982]	0.411 [0.317-0.510]	2.138 [1.732-2.639]	0.176 [0.076-0.408]
IL-10 CD14 NGAL calprotectin	199	0.882 [0.822-0.941]	0.868	0.684 [0.561-0.820]	0.659	0.898	0.627 [0.544-0.704]	0.949 [0.886-0.983]	0.440 [0.341-0.543]	2.405 [1.915-3.021]	0.163 [0.070-0.377]
IL-10 PTX3 LBP calprotectin	199	0.873 [0.810-0.935]	0.856	0.684 [0.636-0.758]	0.673	0.898	0.480 [0.398-0.563]	0.935 [0.855-0.979]	0.361 [0.276-0.453]	1.727 [1.442-2.068]	0.213 [0.091-0.496]
IL-6 PCT IP-10 gelsolin	199	0.879 [0.820-0.938]	0.867	0.683 [0.625-0.784]	0.663	0.898	0.533 [0.450-0.615]	0.941 [0.868-0.981]	0.386 [0.296-0.482]	1.924 [1.583-2.339]	0.191 [0.082-0.445]
IP-10 IL-10 CD14 gelsolin	199	0.873 [0.813-0.934]	0.866	0.682 [0.576-0.816]	0.673	0.898	0.647 [0.565-0.723]	0.951 [0.889-0.984]	0.454 [0.352-0.558]	2.541 [2.007-3.218]	0.158 [0.068-0.365]
PCT PTX3 CD14 NGAL	202	0.845 [0.783-0.907]	0.819	0.681 [0.570-0.825]	0.67	0.898	0.647 [0.566-0.723]	0.952 [0.891-0.984]	0.449 [0.348-0.553]	2.544 [2.013-3.216]	0.158 [0.068-0.365]
IP-10 IL-10 LBP NGAL	202	0.885 [0.826-0.944]	0.875	0.681 [0.594-0.812]	0.677	0.898	0.588 [0.506-0.667]	0.947 [0.881-0.983]	0.411 [0.317-0.510]	2.181 [1.765-2.695]	0.173 [0.075-0.402]
PCT CD14 NGAL gelsolin	199	0.852 [0.791-0.913]	0.835	0.680 [0.593-0.806]	0.667	0.898	0.640 [0.558-0.717]	0.950 [0.888-0.984]	0.449 [0.348-0.553]	2.494 [1.975-3.150]	0.159 [0.069-0.369]
IL-6 PCT LBP gelsolin	199	0.876 [0.816-0.935]	0.860	0.680 [0.626-0.786]	0.656	0.898	0.547 [0.463-0.628]	0.943 [0.871-0.981]	0.393 [0.302-0.490]	1.981 [1.623-2.418]	0.187 [0.080-0.434]
IL-6 PTX3 NGAL calprotectin	199	0.881 [0.822-0.939]	0.862	0.680 [0.605-0.813]	0.663	0.898	0.633 [0.551-0.710]	0.950 [0.887-0.984]	0.444 [0.345-0.548]	2.449 [1.945-3.084]	0.161 [0.070-0.373]
IL-6 IL-10 PTX3 NGAL	202	0.890 [0.833-0.947]	0.878	0.680 [0.617-0.821]	0.663	0.898	0.621 [0.539-0.698]	0.950 [0.887-0.984]	0.431 [0.334-0.533]	2.369 [1.894-2.963]	0.164 [0.071-0.381]
IL-10 PTX3 CD14 LBP	202	0.877 [0.817-0.937]	0.865	0.680 [0.628-0.785]	0.682	0.898	0.497 [0.415-0.579]	0.938 [0.862-0.980]	0.364 [0.278-0.456]	1.784 [1.485-2.144]	0.205 [0.088-0.479]
IP-10 IL-10 NGAL calprotectin	199	0.882 [0.823-0.941]	0.868	0.679 [0.572-0.822]	0.654	0.898	0.653 [0.571-0.729]	0.951 [0.890-0.984]	0.458 [0.356-0.563]	2.590 [2.039-3.290]	0.156 [0.068-0.361]
IL-6 PTX3 LBP NGAL	202	0.880 [0.821-0.939]	0.864	0.678 [0.604-0.815]	0.674	0.898	0.641 [0.559-0.716]	0.951 [0.890-0.984]	0.444 [0.345-0.548]	2.498 [1.982-3.149]	0.159 [0.069-0.369]
IP-10 IL-10 PTX3 CD14	202	0.864 [0.801-0.928]	0.849	0.678 [0.605-0.767]	0.669	0.898	0.510 [0.428-0.591]	0.940 [0.865-0.980]	0.370 [0.283-0.463]	1.832 [1.519-2.209]	0.200 [0.086-0.466]
IL-6 PCT CD14 NGAL	202	0.883 [0.824-0.942]	0.871	0.677 [0.591-0.816]	0.658	0.898	0.660 [0.579-0.735]	0.953 [0.893-0.985]	0.458 [0.356-0.563]	2.642 [2.078-3.359]	0.155 [0.067-0.357]
IL-6 PTX3 calprotectin gelsolin	199	0.869 [0.807-0.930]	0.856	0.677 [0.614-0.776]	0.662	0.898	0.553 [0.470-0.634]	0.943 [0.872-0.981]	0.396 [0.305-0.494]	2.010 [1.643-2.459]	0.184 [0.079-0.428]
IL-6 PCT IL-10 calprotectin	201	0.893 [0.836-0.949]	0.879	0.677 [0.604-0.817]	0.66	0.902	0.560 [0.477-0.641]	0.944 [0.874-0.982]	0.411 [0.319-0.508]	2.050 [1.675-2.509]	0.175 [0.075-0.407]

IL-6 PCT IL-10 LBP	202	0.890 [0.833-0.947]	0.878	0.677 [0.619-0.818]	0.658	0.898	0.588 [0.506-0.667]	0.947 [0.881-0.983]	0.411 [0.317-0.510]	2.181 [1.765-2.695]	0.173 [0.075-0.402]
LBP NGAL calprotectin gelsolin	199	0.847 [0.784-0.910]	0.829	0.677 [0.582-0.809]	0.669	0.898	0.647 [0.565-0.723]	0.951 [0.889-0.984]	0.454 [0.352-0.558]	2.541 [2.007-3.218]	0.158 [0.068-0.365]
PCT IL-10 PTX3 calprotectin	199	0.875 [0.815-0.935]	0.856	0.677 [0.635-0.784]	0.653	0.898	0.580 [0.497-0.660]	0.946 [0.878-0.982]	0.411 [0.317-0.510]	2.138 [1.732-2.639]	0.176 [0.076-0.408]
IL-6 PCT IL-10 NGAL	202	0.896 [0.839-0.953]	0.885	0.676 [0.596-0.846]	0.646	0.898	0.641 [0.559-0.716]	0.951 [0.890-0.984]	0.444 [0.345-0.548]	2.498 [1.982-3.149]	0.159 [0.069-0.369]
IP-10 PTX3 CD14 NGAL	202	0.858 [0.795-0.920]	0.838	0.676 [0.536-0.835]	0.611	0.898	0.693 [0.613-0.765]	0.955 [0.898-0.985]	0.484 [0.377-0.591]	2.923 [2.263-3.776]	0.147 [0.064-0.340]
IP-10 IL-10 calprotectin gelsolin	199	0.874 [0.814-0.935]	0.860	0.676 [0.603-0.800]	0.655	0.898	0.553 [0.470-0.634]	0.943 [0.872-0.981]	0.396 [0.305-0.494]	2.010 [1.643-2.459]	0.184 [0.079-0.428]
IL-6 IL-10 LBP gelsolin	199	0.883 [0.824-0.943]	0.870	0.675 [0.603-0.798]	0.653	0.898	0.500 [0.417-0.583]	0.938 [0.860-0.979]	0.370 [0.283-0.463]	1.796 [1.491-2.163]	0.204 [0.088-0.476]
IL-6 IP-10 PTX3 NGAL	202	0.880 [0.820-0.939]	0.862	0.674 [0.593-0.815]	0.652	0.898	0.641 [0.559-0.716]	0.951 [0.890-0.984]	0.444 [0.345-0.548]	2.498 [1.982-3.149]	0.159 [0.069-0.369]
IL-6 PCT NGAL calprotectin	199	0.881 [0.821-0.941]	0.866	0.674 [0.581-0.812]	0.64	0.898	0.640 [0.558-0.717]	0.950 [0.888-0.984]	0.449 [0.348-0.553]	2.494 [1.975-3.150]	0.159 [0.069-0.369]
IL-6 PCT LBP NGAL	202	0.880 [0.821-0.940]	0.867	0.673 [0.585-0.810]	0.66	0.898	0.634 [0.552-0.710]	0.951 [0.889-0.984]	0.440 [0.341-0.543]	2.453 [1.951-3.084]	0.161 [0.070-0.373]
IL-6 IL-10 calprotectin gelsolin	199	0.883 [0.824-0.942]	0.869	0.672 [0.599-0.811]	0.648	0.898	0.593 [0.510-0.673]	0.947 [0.880-0.983]	0.419 [0.323-0.519]	2.208 [1.781-2.738]	0.172 [0.074-0.399]
IP-10 IL-10 PTX3 calprotectin	199	0.864 [0.800-0.928]	0.851	0.672 [0.622-0.757]	0.657	0.898	0.453 [0.372-0.537]	0.932 [0.847-0.977]	0.349 [0.266-0.439]	1.643 [1.381-1.954]	0.225 [0.096-0.526]
IL-10 PTX3 CD14 calprotectin	199	0.866 [0.802-0.929]	0.851	0.671 [0.623-0.763]	0.661	0.898	0.493 [0.411-0.576]	0.937 [0.858-0.979]	0.367 [0.281-0.459]	1.772 [1.474-2.130]	0.207 [0.089-0.482]
PCT IP-10 IL-10 CD14	202	0.878 [0.819-0.937]	0.861	0.671 [0.616-0.811]	0.645	0.898	0.588 [0.506-0.667]	0.947 [0.881-0.983]	0.411 [0.317-0.510]	2.181 [1.765-2.695]	0.173 [0.075-0.402]
IP-10 IL-10 CD14 LBP	202	0.874 [0.812-0.935]	0.866	0.671 [0.619-0.780]	0.671	0.898	0.529 [0.447-0.611]	0.942 [0.870-0.981]	0.379 [0.291-0.474]	1.908 [1.574-2.314]	0.193 [0.083-0.448]
IL-6 IL-10 PTX3 CD14	202	0.882 [0.823-0.942]	0.867	0.671 [0.597-0.798]	0.656	0.898	0.608 [0.526-0.686]	0.949 [0.885-0.983]	0.423 [0.327-0.524]	2.290 [1.840-2.850]	0.168 [0.072-0.389]
IL-6 PCT IL-10 CD14	202	0.893 [0.835-0.952]	0.883	0.671 [0.591-0.829]	0.64	0.898	0.569 [0.486-0.648]	0.946 [0.878-0.982]	0.400 [0.308-0.498]	2.082 [1.696-2.555]	0.179 [0.077-0.417]
IL-6 IP-10 IL-10 gelsolin	199	0.886 [0.827-0.946]	0.872	0.670 [0.578-0.823]	0.63	0.898	0.587 [0.503-0.666]	0.946 [0.879-0.982]	0.415 [0.320-0.515]	2.172 [1.756-2.687]	0.174 [0.075-0.403]
IP-10 IL-10 LBP calprotectin	199	0.871 [0.808-0.934]	0.859	0.670 [0.627-0.763]	0.649	0.898	0.453 [0.372-0.537]	0.932 [0.847-0.977]	0.349 [0.266-0.439]	1.643 [1.381-1.954]	0.225 [0.096-0.526]
IL-6 PCT IP-10 NGAL	202	0.881 [0.820-0.941]	0.868	0.670 [0.573-0.817]	0.644	0.898	0.654 [0.573-0.729]	0.952 [0.892-0.984]	0.454 [0.352-0.558]	2.592 [2.045-3.286]	0.156 [0.067-0.361]
IL-6 PCT CD14 calprotectin	199	0.871 [0.809-0.933]	0.860	0.670 [0.587-0.784]	0.635	0.898	0.487 [0.404-0.570]	0.936 [0.857-0.979]	0.364 [0.278-0.456]	1.749 [1.458-2.099]	0.210 [0.090-0.489]
IP-10 PTX3 LBP NGAL	202	0.859 [0.796-0.922]	0.838	0.669 [0.538-0.820]	0.626	0.898	0.647 [0.566-0.723]	0.952 [0.891-0.984]	0.449 [0.348-0.553]	2.544 [2.013-3.216]	0.158 [0.068-0.365]
IL-10 CD14 LBP calprotectin	199	0.870 [0.807-0.934]	0.855	0.669 [0.631-0.749]	0.654	0.898	0.460 [0.378-0.543]	0.932 [0.849-0.978]	0.352 [0.269-0.442]	1.663 [1.396-1.981]	0.222 [0.095-0.518]
IL-6 PTX3 CD14 calprotectin	199	0.867 [0.805-0.930]	0.852	0.668 [0.597-0.769]	0.653	0.898	0.520 [0.437-0.602]	0.940 [0.865-0.980]	0.379 [0.291-0.474]	1.871 [1.545-2.265]	0.196 [0.084-0.457]
IL-6 IL-10 CD14 NGAL	202	0.890 [0.832-0.949]	0.878	0.668 [0.583-0.832]	0.643	0.898	0.654 [0.573-0.729]	0.952 [0.892-0.984]	0.454 [0.352-0.558]	2.592 [2.045-3.286]	0.156 [0.067-0.361]
PCT IP-10 IL-10 calprotectin	201	0.869 [0.809-0.929]	0.853	0.668 [0.616-0.785]	0.655	0.902	0.547 [0.463-0.628]	0.943 [0.871-0.981]	0.404 [0.313-0.499]	1.990 [1.633-2.424]	0.179 [0.077-0.418]
IL-6 IL-10 CD14 gelsolin	199	0.883 [0.823-0.944]	0.870	0.668 [0.579-0.812]	0.628	0.898	0.480 [0.398-0.563]	0.935 [0.855-0.979]	0.361 [0.276-0.453]	1.727 [1.442-2.068]	0.213 [0.091-0.496]
IL-10 LBP NGAL calprotectin	199	0.882 [0.821-0.943]	0.871	0.668 [0.579-0.802]	0.646	0.898	0.593 [0.510-0.673]	0.947 [0.880-0.983]	0.419 [0.323-0.519]	2.208 [1.781-2.738]	0.172 [0.074-0.399]
PCT PTX3 calprotectin gelsolin	199	0.781 [0.712-0.849]	0.751	0.667 [0.622-0.751]	0.661	0.898	0.460 [0.378-0.543]	0.932 [0.849-0.978]	0.352 [0.269-0.442]	1.663 [1.396-1.981]	0.222 [0.095-0.518]
IP-10 LBP NGAL gelsolin	199	0.859 [0.797-0.922]	0.845	0.667 [0.553-0.807]	0.63	0.898	0.620 [0.537-0.698]	0.949 [0.885-0.983]	0.436 [0.337-0.538]	2.363 [1.887-2.960]	0.165 [0.071-0.381]
IL-6 PTX3 CD14 gelsolin	199	0.870 [0.809-0.932]	0.860	0.667 [0.604-0.777]	0.652	0.898	0.580 [0.497-0.660]	0.946 [0.878-0.982]	0.411 [0.317-0.510]	2.138 [1.732-2.639]	0.176 [0.076-0.408]
IL-6 IP-10 IL-10 NGAL	202	0.890 [0.831-0.949]	0.876	0.666 [0.593-0.826]	0.641	0.898	0.595 [0.513-0.673]	0.948 [0.883-0.983]	0.415 [0.320-0.515]	2.216 [1.789-2.744]	0.172 [0.074-0.398]
IL-6 PCT LBP calprotectin	199	0.872 [0.810-0.934]	0.858	0.666 [0.587-0.782]	0.632	0.898	0.547 [0.463-0.628]	0.943 [0.871-0.981]	0.393 [0.302-0.490]	1.981 [1.623-2.418]	0.187 [0.080-0.434]
PCT IP-10 PTX3 gelsolin	199	0.818 [0.752-0.885]	0.800	0.666 [0.579-0.763]	0.646	0.898	0.520 [0.437-0.602]	0.940 [0.865-0.980]	0.379 [0.291-0.474]	1.871 [1.545-2.265]	0.196 [0.084-0.457]

IP-10 NGAL calprotectin gelsolin	199	0.855 [0.791-0.919]	0.843	0.665 [0.539-0.822]	0.62	0.898	0.667 [0.585-0.741]	0.952 [0.892-0.984]	0.468 [0.364-0.574]	2.694 [2.108-3.442]	0.153 [0.066-0.354]
PCT PTX3 LBP gelsolin	199	0.820 [0.755-0.886]	0.794	0.664 [0.603-0.777]	0.652	0.898	0.600 [0.517-0.679]	0.947 [0.881-0.983]	0.423 [0.327-0.524]	2.245 [1.806-2.790]	0.170 [0.073-0.394]
IL-6 PCT IP-10 calprotectin	201	0.876 [0.815-0.936]	0.862	0.663 [0.582-0.790]	0.645	0.902	0.580 [0.497-0.660]	0.946 [0.878-0.982]	0.422 [0.328-0.520]	2.148 [1.743-2.646]	0.169 [0.073-0.393]
IL-6 IP-10 PTX3 calprotectin	199	0.867 [0.803-0.930]	0.849	0.662 [0.591-0.767]	0.643	0.898	0.487 [0.404-0.570]	0.936 [0.857-0.979]	0.364 [0.278-0.456]	1.749 [1.458-2.099]	0.210 [0.090-0.489]
IL-6 IL-10 LBP NGAL	202	0.889 [0.830-0.948]	0.879	0.662 [0.595-0.821]	0.641	0.898	0.627 [0.546-0.704]	0.950 [0.888-0.984]	0.436 [0.337-0.538]	2.410 [1.922-3.022]	0.163 [0.070-0.377]
IL-6 IL-10 PTX3 calprotectin	199	0.878 [0.816-0.939]	0.864	0.662 [0.599-0.785]	0.63	0.898	0.527 [0.444-0.609]	0.940 [0.867-0.980]	0.383 [0.294-0.478]	1.897 [1.563-2.302]	0.194 [0.083-0.451]
IL-6 CD14 NGAL calprotectin	199	0.873 [0.811-0.936]	0.860	0.661 [0.568-0.799]	0.63	0.898	0.593 [0.510-0.673]	0.947 [0.880-0.983]	0.419 [0.323-0.519]	2.208 [1.781-2.738]	0.172 [0.074-0.399]
IL-6 IL-10 PTX3 LBP	202	0.882 [0.822-0.943]	0.869	0.660 [0.594-0.803]	0.641	0.898	0.582 [0.499-0.661]	0.947 [0.880-0.983]	0.407 [0.314-0.506]	2.147 [1.741-2.647]	0.175 [0.076-0.407]
IL-6 CD14 LBP NGAL	202	0.875 [0.813-0.937]	0.861	0.659 [0.577-0.801]	0.642	0.898	0.627 [0.546-0.704]	0.950 [0.888-0.984]	0.436 [0.337-0.538]	2.410 [1.922-3.022]	0.163 [0.070-0.377]
IL-6 IP-10 PTX3 gelsolin	199	0.870 [0.808-0.932]	0.860	0.658 [0.597-0.777]	0.641	0.898	0.580 [0.497-0.660]	0.946 [0.878-0.982]	0.411 [0.317-0.510]	2.138 [1.732-2.639]	0.176 [0.076-0.408]
PCT PTX3 LBP calprotectin	199	0.819 [0.752-0.886]	0.792	0.658 [0.598-0.762]	0.645	0.898	0.587 [0.503-0.666]	0.946 [0.879-0.982]	0.415 [0.320-0.515]	2.172 [1.756-2.687]	0.174 [0.075-0.403]
PCT IP-10 PTX3 CD14	202	0.831 [0.764-0.897]	0.807	0.658 [0.562-0.762]	0.629	0.898	0.477 [0.396-0.559]	0.936 [0.857-0.979]	0.355 [0.271-0.446]	1.717 [1.437-2.053]	0.214 [0.092-0.499]
IL-6 IP-10 IL-10 LBP	202	0.882 [0.821-0.943]	0.870	0.657 [0.594-0.809]	0.627	0.898	0.523 [0.441-0.604]	0.941 [0.868-0.981]	0.376 [0.288-0.470]	1.882 [1.555-2.278]	0.195 [0.084-0.454]
IL-6 IP-10 CD14 NGAL	202	0.873 [0.810-0.936]	0.862	0.657 [0.561-0.802]	0.627	0.898	0.601 [0.519-0.679]	0.948 [0.884-0.983]	0.419 [0.323-0.519]	2.252 [1.814-2.796]	0.170 [0.073-0.393]
PCT CD14 LBP NGAL	202	0.839 [0.773-0.905]	0.818	0.657 [0.565-0.796]	0.65	0.898	0.608 [0.526-0.686]	0.949 [0.885-0.983]	0.423 [0.327-0.524]	2.290 [1.840-2.850]	0.168 [0.072-0.389]
IP-10 CD14 NGAL gelsolin	199	0.857 [0.792-0.921]	0.845	0.657 [0.537-0.815]	0.604	0.898	0.640 [0.558-0.717]	0.950 [0.888-0.984]	0.449 [0.348-0.553]	2.494 [1.975-3.150]	0.159 [0.069-0.369]
IL-6 PCT CD14 LBP	202	0.872 [0.809-0.935]	0.860	0.657 [0.582-0.780]	0.627	0.898	0.516 [0.434-0.598]	0.940 [0.867-0.980]	0.373 [0.286-0.467]	1.857 [1.537-2.243]	0.198 [0.085-0.460]
IL-6 PTX3 LBP calprotectin	199	0.869 [0.805-0.932]	0.851	0.656 [0.593-0.767]	0.634	0.898	0.553 [0.470-0.634]	0.943 [0.872-0.981]	0.396 [0.305-0.494]	2.010 [1.643-2.459]	0.184 [0.079-0.428]
PCT IL-10 CD14 calprotectin	199	0.874 [0.812-0.936]	0.858	0.655 [0.598-0.787]	0.623	0.898	0.553 [0.470-0.634]	0.943 [0.872-0.981]	0.396 [0.305-0.494]	2.010 [1.643-2.459]	0.184 [0.079-0.428]
IL-6 LBP NGAL calprotectin	199	0.874 [0.811-0.937]	0.858	0.655 [0.560-0.800]	0.624	0.898	0.607 [0.524-0.685]	0.948 [0.883-0.983]	0.427 [0.330-0.528]	2.283 [1.832-2.845]	0.168 [0.073-0.390]
IL-6 IL-10 NGAL calprotectin	199	0.887 [0.827-0.948]	0.876	0.655 [0.572-0.820]	0.612	0.898	0.620 [0.537-0.698]	0.949 [0.885-0.983]	0.436 [0.337-0.538]	2.363 [1.887-2.960]	0.165 [0.071-0.381]
IL-6 PTX3 CD14 LBP	202	0.869 [0.805-0.932]	0.860	0.654 [0.591-0.772]	0.628	0.898	0.529 [0.447-0.611]	0.942 [0.870-0.981]	0.379 [0.291-0.474]	1.908 [1.574-2.314]	0.193 [0.083-0.448]
IL-6 PTX3 LBP gelsolin	199	0.870 [0.807-0.933]	0.852	0.653 [0.599-0.774]	0.63	0.898	0.540 [0.457-0.622]	0.942 [0.870-0.981]	0.389 [0.299-0.486]	1.952 [1.602-2.378]	0.189 [0.081-0.439]
PCT IP-10 PTX3 LBP	202	0.845 [0.778-0.911]	0.824	0.653 [0.550-0.773]	0.638	0.898	0.562 [0.480-0.642]	0.945 [0.876-0.982]	0.396 [0.305-0.494]	2.051 [1.674-2.512]	0.182 [0.078-0.421]
IL-6 IP-10 LBP NGAL	202	0.874 [0.810-0.938]	0.860	0.652 [0.556-0.802]	0.619	0.898	0.627 [0.546-0.704]	0.950 [0.888-0.984]	0.436 [0.337-0.538]	2.410 [1.922-3.022]	0.163 [0.070-0.377]
IL-6 PCT IP-10 LBP	202	0.871 [0.807-0.935]	0.861	0.651 [0.574-0.777]	0.614	0.898	0.451 [0.371-0.533]	0.932 [0.849-0.978]	0.344 [0.262-0.433]	1.636 [1.377-1.942]	0.226 [0.097-0.529]
PTX3 CD14 LBP gelsolin	199	0.816 [0.747-0.885]	0.798	0.651 [0.571-0.759]	0.638	0.898	0.547 [0.463-0.628]	0.943 [0.871-0.981]	0.393 [0.302-0.490]	1.981 [1.623-2.418]	0.187 [0.080-0.434]
IL-6 PCT IP-10 CD14	202	0.873 [0.809-0.936]	0.862	0.650 [0.578-0.780]	0.619	0.898	0.549 [0.467-0.629]	0.944 [0.874-0.982]	0.389 [0.299-0.486]	1.991 [1.632-2.429]	0.186 [0.080-0.432]
CD14 NGAL calprotectin gelsolin	199	0.847 [0.783-0.912]	0.830	0.650 [0.556-0.811]	0.621	0.898	0.667 [0.585-0.741]	0.952 [0.892-0.984]	0.468 [0.364-0.574]	2.694 [2.108-3.442]	0.153 [0.066-0.354]
PTX3 CD14 LBP calprotectin	199	0.818 [0.749-0.887]	0.794	0.649 [0.569-0.761]	0.633	0.898	0.560 [0.477-0.641]	0.944 [0.874-0.982]	0.400 [0.308-0.498]	2.041 [1.665-2.502]	0.182 [0.078-0.423]
PCT PTX3 CD14 LBP	202	0.820 [0.752-0.888]	0.798	0.649 [0.587-0.752]	0.647	0.898	0.497 [0.415-0.579]	0.938 [0.862-0.980]	0.364 [0.278-0.456]	1.784 [1.485-2.144]	0.205 [0.088-0.479]
IL-6 IP-10 PTX3 CD14	202	0.867 [0.803-0.931]	0.858	0.649 [0.581-0.771]	0.621	0.898	0.549 [0.467-0.629]	0.944 [0.874-0.982]	0.389 [0.299-0.486]	1.991 [1.632-2.429]	0.186 [0.080-0.432]
IL-6 CD14 calprotectin gelsolin	199	0.861 [0.795-0.926]	0.846	0.647 [0.579-0.762]	0.614	0.898	0.480 [0.398-0.563]	0.935 [0.855-0.979]	0.361 [0.276-0.453]	1.727 [1.442-2.068]	0.213 [0.091-0.496]
IL-6 IL-10 LBP calprotectin	199	0.878 [0.815-0.941]	0.863	0.647 [0.594-0.787]	0.61	0.898	0.487 [0.404-0.570]	0.936 [0.857-0.979]	0.364 [0.278-0.456]	1.749 [1.458-2.099]	0.210 [0.090-0.489]

IL-6 IP-10 IL-10 CD14	202	0.881 [0.818-0.944]	0.867	0.646 [0.556-0.814]	0.608	0.898	0.484 [0.402-0.566]	0.937 [0.858-0.979]	0.358 [0.273-0.449]	1.739 [1.452-2.082]	0.211 [0.090-0.492]
IP-10 IL-10 CD14 calprotectin	199	0.862 [0.796-0.928]	0.850	0.646 [0.584-0.760]	0.618	0.898	0.480 [0.398-0.563]	0.935 [0.855-0.979]	0.361 [0.276-0.453]	1.727 [1.442-2.068]	0.213 [0.091-0.496]
IL-6 IL-10 CD14 LBP	202	0.881 [0.819-0.943]	0.866	0.645 [0.584-0.806]	0.63	0.898	0.641 [0.559-0.716]	0.951 [0.890-0.984]	0.444 [0.345-0.548]	2.498 [1.982-3.149]	0.159 [0.069-0.369]
IL-6 IP-10 IL-10 calprotectin	201	0.875 [0.812-0.938]	0.854	0.644 [0.576-0.778]	0.619	0.902	0.493 [0.411-0.576]	0.937 [0.858-0.979]	0.377 [0.291-0.469]	1.780 [1.484-2.136]	0.199 [0.085-0.464]
IP-10 PTX3 CD14 calprotectin	199	0.815 [0.744-0.885]	0.796	0.644 [0.554-0.744]	0.627	0.898	0.507 [0.424-0.589]	0.938 [0.862-0.980]	0.373 [0.286-0.467]	1.820 [1.509-2.196]	0.201 [0.086-0.469]
PCT LBP calprotectin gelsolin	199	0.813 [0.743-0.882]	0.794	0.643 [0.550-0.757]	0.615	0.898	0.513 [0.430-0.596]	0.939 [0.863-0.980]	0.376 [0.288-0.470]	1.845 [1.527-2.230]	0.199 [0.085-0.463]
CD14 LBP NGAL calprotectin	199	0.835 [0.767-0.903]	0.814	0.643 [0.539-0.794]	0.609	0.898	0.613 [0.530-0.692]	0.948 [0.884-0.983]	0.431 [0.334-0.533]	2.322 [1.859-2.901]	0.166 [0.072-0.385]
PCT LBP NGAL calprotectin	199	0.835 [0.768-0.903]	0.810	0.643 [0.544-0.788]	0.621	0.898	0.600 [0.517-0.679]	0.947 [0.881-0.983]	0.423 [0.327-0.524]	2.245 [1.806-2.790]	0.170 [0.073-0.394]
PCT IP-10 LBP NGAL	202	0.854 [0.787-0.920]	0.839	0.643 [0.526-0.797]	0.575	0.898	0.608 [0.526-0.686]	0.949 [0.885-0.983]	0.423 [0.327-0.524]	2.290 [1.840-2.850]	0.168 [0.072-0.389]
IL-6 IP-10 NGAL calprotectin	199	0.872 [0.806-0.937]	0.858	0.643 [0.540-0.801]	0.602	0.898	0.600 [0.517-0.679]	0.947 [0.881-0.983]	0.423 [0.327-0.524]	2.245 [1.806-2.790]	0.170 [0.073-0.394]
IL-6 LBP calprotectin gelsolin	199	0.861 [0.794-0.927]	0.847	0.641 [0.574-0.756]	0.604	0.898	0.493 [0.411-0.576]	0.937 [0.858-0.979]	0.367 [0.281-0.459]	1.772 [1.474-2.130]	0.207 [0.089-0.482]
IP-10 PTX3 calprotectin gelsolin	199	0.798 [0.726-0.870]	0.775	0.639 [0.586-0.729]	0.639	0.898	0.453 [0.372-0.537]	0.932 [0.847-0.977]	0.349 [0.266-0.439]	1.643 [1.381-1.954]	0.225 [0.096-0.526]
IL-6 IL-10 CD14 calprotectin	199	0.875 [0.811-0.939]	0.860	0.639 [0.581-0.784]	0.59	0.898	0.453 [0.372-0.537]	0.932 [0.847-0.977]	0.349 [0.266-0.439]	1.643 [1.381-1.954]	0.225 [0.096-0.526]
PTX3 LBP calprotectin gelsolin	199	0.815 [0.746-0.884]	0.794	0.638 [0.559-0.765]	0.615	0.898	0.580 [0.497-0.660]	0.946 [0.878-0.982]	0.411 [0.317-0.510]	2.138 [1.732-2.639]	0.176 [0.076-0.408]
IL-6 IP-10 PTX3 LBP	202	0.868 [0.803-0.932]	0.852	0.638 [0.580-0.773]	0.605	0.898	0.595 [0.513-0.673]	0.948 [0.883-0.983]	0.415 [0.320-0.515]	2.216 [1.789-2.744]	0.172 [0.074-0.398]
IL-6 CD14 LBP calprotectin	199	0.859 [0.791-0.927]	0.842	0.637 [0.559-0.752]	0.587	0.898	0.453 [0.372-0.537]	0.932 [0.847-0.977]	0.349 [0.266-0.439]	1.643 [1.381-1.954]	0.225 [0.096-0.526]
IL-6 IP-10 calprotectin gelsolin	199	0.860 [0.794-0.926]	0.847	0.636 [0.588-0.760]	0.61	0.898	0.467 [0.385-0.550]	0.933 [0.851-0.978]	0.355 [0.271-0.446]	1.684 [1.411-2.010]	0.219 [0.094-0.511]
PCT PTX3 CD14 gelsolin	199	0.814 [0.744-0.884]	0.785	0.635 [0.540-0.756]	0.572	0.898	0.513 [0.430-0.596]	0.939 [0.863-0.980]	0.376 [0.288-0.470]	1.845 [1.527-2.230]	0.199 [0.085-0.463]
IP-10 CD14 LBP NGAL	202	0.853 [0.786-0.921]	0.839	0.633 [0.521-0.792]	0.553	0.898	0.588 [0.506-0.667]	0.947 [0.881-0.983]	0.411 [0.317-0.510]	2.181 [1.765-2.695]	0.173 [0.075-0.402]
PCT IP-10 NGAL calprotectin	199	0.848 [0.781-0.916]	0.834	0.630 [0.514-0.805]	0.538	0.898	0.633 [0.551-0.710]	0.950 [0.887-0.984]	0.444 [0.345-0.548]	2.449 [1.945-3.084]	0.161 [0.070-0.373]
IL-6 CD14 LBP gelsolin	199	0.861 [0.793-0.929]	0.850	0.627 [0.553-0.757]	0.571	0.898	0.467 [0.385-0.550]	0.933 [0.851-0.978]	0.355 [0.271-0.446]	1.684 [1.411-2.010]	0.219 [0.094-0.511]
IP-10 PTX3 CD14 gelsolin	199	0.814 [0.742-0.885]	0.794	0.626 [0.533-0.743]	0.607	0.898	0.473 [0.391-0.556]	0.934 [0.853-0.978]	0.358 [0.273-0.449]	1.705 [1.426-2.039]	0.216 [0.092-0.503]
PTX3 CD14 calprotectin gelsolin	199	0.792 [0.719-0.864]	0.761	0.624 [0.565-0.723]	0.602	0.898	0.433 [0.353-0.517]	0.929 [0.841-0.976]	0.341 [0.260-0.430]	1.585 [1.339-1.876]	0.235 [0.101-0.551]
PCT PTX3 CD14 calprotectin	199	0.805 [0.734-0.876]	0.782	0.624 [0.547-0.745]	0.569	0.898	0.500 [0.417-0.583]	0.938 [0.860-0.979]	0.370 [0.283-0.463]	1.796 [1.491-2.163]	0.204 [0.088-0.476]
IL-6 IP-10 CD14 calprotectin	199	0.856 [0.786-0.925]	0.844	0.624 [0.554-0.747]	0.577	0.898	0.460 [0.378-0.543]	0.932 [0.849-0.978]	0.352 [0.269-0.442]	1.663 [1.396-1.981]	0.222 [0.095-0.518]
IP-10 LBP NGAL calprotectin	199	0.850 [0.781-0.918]	0.838	0.623 [0.515-0.790]	0.537	0.898	0.567 [0.483-0.647]	0.944 [0.875-0.982]	0.404 [0.311-0.502]	2.072 [1.687-2.546]	0.180 [0.078-0.418]
IL-6 IP-10 LBP calprotectin	199	0.857 [0.787-0.927]	0.846	0.622 [0.549-0.742]	0.556	0.898	0.367 [0.290-0.449]	0.917 [0.816-0.972]	0.317 [0.240-0.401]	1.418 [1.215-1.654]	0.278 [0.118-0.656]
IP-10 PTX3 CD14 LBP	202	0.836 [0.765-0.906]	0.820	0.620 [0.531-0.757]	0.581	0.898	0.542 [0.460-0.623]	0.943 [0.872-0.981]	0.386 [0.296-0.482]	1.963 [1.612-2.389]	0.188 [0.081-0.437]
PCT IP-10 LBP calprotectin	199	0.833 [0.761-0.904]	0.811	0.617 [0.524-0.755]	0.575	0.898	0.507 [0.424-0.589]	0.938 [0.862-0.980]	0.373 [0.286-0.467]	1.820 [1.509-2.196]	0.201 [0.086-0.469]
PCT CD14 LBP calprotectin	199	0.806 [0.732-0.880]	0.777	0.616 [0.534-0.736]	0.572	0.898	0.487 [0.404-0.570]	0.936 [0.857-0.979]	0.364 [0.278-0.456]	1.749 [1.458-2.099]	0.210 [0.090-0.489]
PCT CD14 NGAL calprotectin	199	0.830 [0.760-0.900]	0.816	0.616 [0.528-0.777]	0.573	0.898	0.567 [0.483-0.647]	0.944 [0.875-0.982]	0.404 [0.311-0.502]	2.072 [1.687-2.546]	0.180 [0.078-0.418]
PCT IP-10 CD14 NGAL	202	0.851 [0.782-0.919]	0.830	0.616 [0.508-0.795]	0.54	0.898	0.647 [0.566-0.723]	0.952 [0.891-0.984]	0.449 [0.348-0.553]	2.544 [2.013-3.216]	0.158 [0.068-0.365]
IL-6 IP-10 CD14 gelsolin	199	0.859 [0.790-0.928]	0.846	0.614 [0.553-0.756]	0.562	0.898	0.493 [0.411-0.576]	0.937 [0.858-0.979]	0.367 [0.281-0.459]	1.772 [1.474-2.130]	0.207 [0.089-0.482]
IL-6 IP-10 CD14 LBP	202	0.860 [0.790-0.930]	0.855	0.612 [0.540-0.754]	0.555	0.898	0.418 [0.339-0.501]	0.928 [0.839-0.976]	0.331 [0.252-0.418]	1.544 [1.310-1.819]	0.244 [0.104-0.572]

IP-10 PTX3 LBP calprotectin	199	0.832 [0.761-0.904]	0.812	0.612 [0.541-0.748]	0.581	0.898	0.467 [0.385-0.550]	0.933 [0.851-0.978]	0.355 [0.271-0.446]	1.684 [1.411-2.010]	0.219 [0.094-0.511]
CD14 LBP calprotectin gelsolin	199	0.800 [0.727-0.874]	0.774	0.611 [0.537-0.735]	0.549	0.898	0.473 [0.391-0.556]	0.934 [0.853-0.978]	0.358 [0.273-0.449]	1.705 [1.426-2.039]	0.216 [0.092-0.503]
PCT IP-10 CD14 gelsolin	199	0.815 [0.740-0.889]	0.798	0.608 [0.531-0.711]	0.588	0.898	0.420 [0.340-0.503]	0.926 [0.837-0.976]	0.336 [0.256-0.424]	1.548 [1.312-1.827]	0.243 [0.104-0.569]
IL-6 IP-10 LBP gelsolin	199	0.861 [0.792-0.930]	0.851	0.608 [0.545-0.757]	0.536	0.898	0.473 [0.391-0.556]	0.934 [0.853-0.978]	0.358 [0.273-0.449]	1.705 [1.426-2.039]	0.216 [0.092-0.503]
PCT CD14 LBP gelsolin	199	0.804 [0.730-0.878]	0.781	0.608 [0.511-0.745]	0.547	0.898	0.527 [0.444-0.609]	0.940 [0.867-0.980]	0.383 [0.294-0.478]	1.897 [1.563-2.302]	0.194 [0.083-0.451]
PCT CD14 calprotectin gelsolin	199	0.791 [0.717-0.865]	0.767	0.606 [0.547-0.733]	0.564	0.898	0.360 [0.283-0.442]	0.915 [0.813-0.972]	0.314 [0.239-0.398]	1.403 [1.204-1.635]	0.283 [0.120-0.668]
IP-10 CD14 NGAL calprotectin	199	0.846 [0.776-0.916]	0.830	0.604 [0.506-0.792]	0.499	0.898	0.613 [0.530-0.692]	0.948 [0.884-0.983]	0.431 [0.334-0.533]	2.322 [1.859-2.901]	0.166 [0.072-0.385]
IP-10 PTX3 LBP gelsolin	199	0.827 [0.754-0.900]	0.806	0.602 [0.539-0.742]	0.573	0.898	0.460 [0.378-0.543]	0.932 [0.849-0.978]	0.352 [0.269-0.442]	1.663 [1.396-1.981]	0.222 [0.095-0.518]
PCT IP-10 LBP gelsolin	199	0.835 [0.764-0.907]	0.813	0.597 [0.526-0.767]	0.546	0.898	0.547 [0.463-0.628]	0.943 [0.871-0.981]	0.393 [0.302-0.490]	1.981 [1.623-2.418]	0.187 [0.080-0.434]
IP-10 LBP calprotectin gelsolin	199	0.821 [0.745-0.897]	0.798	0.592 [0.516-0.729]	0.532	0.898	0.433 [0.353-0.517]	0.929 [0.841-0.976]	0.341 [0.260-0.430]	1.585 [1.339-1.876]	0.235 [0.101-0.551]
PCT IP-10 CD14 calprotectin	199	0.819 [0.746-0.893]	0.803	0.588 [0.531-0.748]	0.521	0.898	0.480 [0.398-0.563]	0.935 [0.855-0.979]	0.361 [0.276-0.453]	1.727 [1.442-2.068]	0.213 [0.091-0.496]
IP-10 CD14 LBP calprotectin	199	0.823 [0.746-0.899]	0.810	0.587 [0.489-0.739]	0.5	0.898	0.480 [0.398-0.563]	0.935 [0.855-0.979]	0.361 [0.276-0.453]	1.727 [1.442-2.068]	0.213 [0.091-0.496]
IP-10 CD14 calprotectin gelsolin	199	0.804 [0.727-0.881]	0.784	0.576 [0.508-0.733]	0.517	0.898	0.467 [0.385-0.550]	0.933 [0.851-0.978]	0.355 [0.271-0.446]	1.684 [1.411-2.010]	0.219 [0.094-0.511]
PCT IP-10 CD14 LBP	202	0.829 [0.753-0.905]	0.815	0.569 [0.492-0.755]	0.456	0.898	0.588 [0.506-0.667]	0.947 [0.881-0.983]	0.411 [0.317-0.510]	2.181 [1.765-2.695]	0.173 [0.075-0.402]
IP-10 CD14 LBP gelsolin	199	0.816 [0.737-0.895]	0.804	0.563 [0.495-0.717]	0.469	0.898	0.447 [0.366-0.530]	0.931 [0.845-0.977]	0.346 [0.264-0.436]	1.623 [1.366-1.927]	0.228 [0.098-0.534]

Abbreviations: AUC, area under the curve; CI, confidence interval; NPV, negative predictive value; PPV, positive predictive value; LR-, negative likelihood ratio; LR+, positive likelihood ratio
